# Finite sample size errors in the context of multiple error sources in quantitative medical imaging: An evaluation for breast magnetic resonance diffusion-weighted imaging

**DOI:** 10.1371/journal.pone.0341201

**Published:** 2026-06-04

**Authors:** Jessica V. Eberle, Sebastian Bickelhaupt, Lorenz A. Kapsner, Sabine Ohlmeyer, Evelyn Wenkel, Michael Uder, Dominika Skwierawska, Katharina Tkotz, Dominique Hadler, Tristan A. Kuder, Frederik B. Laun

**Affiliations:** 1 Institute of Radiology, Universitätsklinikum Erlangen, Friedrich-Alexander-Universität Erlangen-Nürnberg (FAU), Erlangen, Germany; 2 German Cancer Research Center (DKFZ), Medical Imaging and Radiology - Cancer Prevention, Heidelberg, Germany; 3 Friedrich-Alexander-Universität Erlangen-Nürnberg (FAU), Medical Informatics, Erlangen, Germany; 4 German Cancer Research Center (DKFZ), Medical Physics in Radiology, Heidelberg, Germany; Medical University of Vienna, AUSTRIA

## Abstract

**Background:**

Selecting appropriate sample sizes in magnetic resonance imaging studies is a complex process that requires to balance statistical rigor with the practical challenges of measuring a large patient population. In this Institutional Review Board approved study, we evaluate the dominant error types (“finite N” errors versus precision errors) for apparent diffusion coefficient (ADC)-based lesion characterization in diffusion-weighted magnetic resonance imaging (DWI) of the female breast in a local dataset and compare our results with current literature.

**Methods:**

First, in a literature review including 24 published breast DWI studies, the standard error of the area under the receiver operating characteristic curve as a measure of sample size-related errors (finite N errors) was estimated for the reported ADC values and compared to the values, derived from expert readings of a university hospital’s cohort of 171 patients with suspicious breast lesions. Second, precision errors were assessed based on published analyses of the coefficient of variation of ADC values, measured in breast DWI exams.

**Results:**

Finite N errors were dominant in the in-house study and most of the 24 reviewed studies. The median sample size at which finite N errors and precision errors were equal was determined to be n = 932.

**Discussion:**

This analysis of dominant error types shows that the required sample sizes for the considered use case are not unreasonably large and that reducing sample sizes may not be justified based on the merits of the conducted analysis. Nonetheless, incorporating dominant error type assessments into future studies may provide valuable insights for optimizing study design and improving methodological rigor.

## Introduction

Choosing an adequate sample size is a key task in research, whether for planning a study, obtaining institutional review board approval, or during the publication review process. Established methods to determine an adequate sample size are often based on (estimated) effect sizes, the desired significance level, and statistical power. These methods are well established and widely used in research; however, they also have limitations. For example, effect sizes may not be known a priori, and there are no strict rules on how to choose the significance level [1–3]. A standard level for the significance threshold is 0.05, but there are also reasons to choose other values, such as 0.005 [4].

Given this uncertainty, examining established practices may provide useful guidance. In the field of magnetic resonance imaging (MRI) research, for example, Hanspach et al. and Bögerl et al. investigated the sample sizes in methodological and clinical MRI studies, with median sample sizes of n = 6 [2] and n = 74 [5], respectively. While these provided descriptive information, they did not assess the suitability of the sample sizes used. To address this limitation, the present study assesses the adequacy of sample sizes, following a methodology common in measurement science – namely, estimating individual uncertainty contributions and identifying which contributes most to the total uncertainty (see Ch. 2–3 of [6]). This can be used to identify the limiting factor in diagnostic performance and guide methodological optimization.

Uncertainty in a quantitative MRI research study may be introduced by using a finite sample size, leading to a “finite N” error. Naturally, further error sources will be present in any study. At a conceptual level, these error types may be classified into accuracy and precision errors. Precision refers to the test-retest-reproducibility, whereas accuracy refers to how close the mean measured quantitative value is to the true value. Generally, accuracy is much harder to assess in quantitative medical imaging studies, where a reliable ground truth is usually missing. As reports on precision are thus generally more readily available, we focused on the comparison of finite N errors and precision errors in the present investigation.

Such an assessment may guide study planning. For example, when the precision error dominates relative to the finite N error, further increasing the sample size may have a limited effect, and efforts may be better directed toward improving measurement precision rather than recruiting additional patients and burdening them with MRI exams.

For our analysis, we chose a use case that we deemed representative of the field – apparent diffusion coefficient (ADC)-based lesion characterization in diffusion-weighted magnetic resonance imaging (DWI) of the female breast, which is an established and relevant application field with sufficient high-quality studies for our analysis to rely on. Usually, the water ADC in malignant breast lesions is lower than in benign lesions, which enables discrimination between the two lesion types [7,8]. A standard approach to assess the clinical value of such quantitative evaluations is receiver operating characteristic (ROC) analysis, which yields the area under the curve (AUC). An AUC of 1 indicates a perfect separation of the two classes, whereas an AUC of 0.5 indicates that the classification performance is statistically not better than random chance. The AUC obtained in a study depends on the specific samples, that is, on the actually measured patients. The standard error of the AUC therefore provides a useful estimate of the finite N error arising from sampling variability. In the present analysis, this error is compared to the precision error, which is derived from studies on the test-retest-reproducibility of breast DWI.

To address this, we applied a methodological framework from physical measurement science that focuses on identifying the dominant source of error. This approach enables us to assess whether finite sample size or measurement imprecision limits diagnostic performance in current practice. Although the present review focuses on ADC-based lesion characterization in breast DWI, the question of whether diagnostic performance is limited by sample size or by measurement precision is relevant across quantitative imaging research. In many applications, diagnostic performance is assessed in the absence of a known ground truth, and characterizing the relative contributions of sampling variability and measurement uncertainty therefore provides critical context for interpreting study results in a broader quantitative imaging setting [9,10].

## Methods

### Data acquisition for the in-house study

This retrospective study was approved by the ethics committee of the Friedrich-Alexander Universität (FAU) Erlangen-Nürnberg, Erlangen, Germany, waiving the need for informed consent. The data for research purposes was accessed from April 1, 2021 until December 31, 2021. The authors were blinded to information that could lead to the identification of individual participants during or after data extraction. Consecutive MRI examinations of n = 359 women from October 2015 to December 2019 were included, reflecting the unbiased routine spectrum of clinically indicated breast MRI examinations.

**Fig 1:**
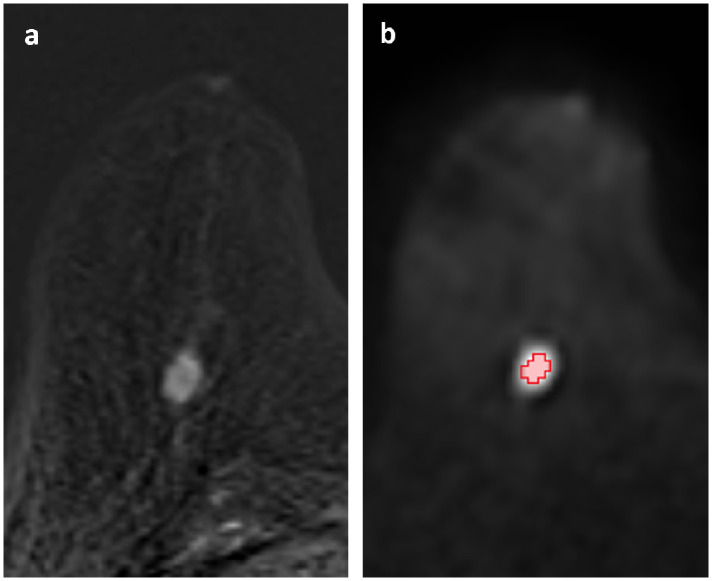
Segmentation example of a malignant breast lesion (in-house study). Example images of a 64-year-old woman with one malignant breast lesion (radiological Breast Imaging Reporting and Data System (BI-RADs) 4, histopathological B5b). **a)** Axial slice from the dynamic contrast-enhanced early subtraction image, an acquisition commonly used in clinical breast MRI to identify enhancing lesions. The subtraction image was generated by subtracting T_1_-weighted MR images acquired before the administration of contrast agent from those acquired after the administration. The malignant breast lesion accumulated contrast agent and thus appears bright in this image. **b)** Corresponding DWI image (b = 1500 s/mm²) with the region of interest (ROI) placed conservatively to minimize partial volume effects. Segmentation volume ≈ 0.2 cm^3^, apparent diffusion coefficient = 0.79 ± 0.12 µm²/ms. The contrast in this image is not generated by a contrast agent. Instead, a diffusion-weighting is applied. The lower the diffusion coefficient, the lower is the signal loss generated by the diffusion-weighting, which leads to a hyperintense signal in this low-diffusivity lesion.

Inclusion and exclusion criteria, details about the histopathological analysis serving as ground truth, the imaging protocol, and the statistical analysis of the in-house study, as well as its limitations, are provided as supporting information (see S1 File). The images were evaluated by a medical student (J.V.E., two years of experience in breast lesion segmentation) who was supervised by a board-certified radiologist (S.B., > 10 years experience in breast MRI). They were not aware of the histopathologic results, but were informed of the BI-RADS classification and the radiology report. Lesions were identified on T_1_-weighted post-contrast subtraction images, taking the radiology report into account. Lesions were manually segmented in 3D Slicer (version 4.11.20210226) on the axial slice of the DWI b = 1500 s/mm² data where they appeared largest (see Fig 1). Boundary voxels that contained fat tissue were excluded from the segmentation.

Histopathology served as ground-truth. 3D Slicer (version 4.11.20210226) was used to calculate the lesion size and the segmentation-averaged ADC values. For the computation of the ADC values, the ADC maps provided by the scanner were used.

To improve the comparability of the ADC values derived from the different publications throughout the literature review, eliminate systematic shifts, and facilitate subsequent statistical simulations, the mean values and standard deviations were normalized according to Equations (1) and (2). The measured $N_{\text{1}}$ data points of class 1 (malignant) and $N_{\text{2}}$ data points of class 2 (benign) are labeled as ${\hat{q}}_{\text{1},n}$ and ${\hat{q}}_{\text{2},n}$, respectively. Here, “q” stands for “quantitative (value)”, i.e., the ADC, for example. The following normalization was performed:


$$\begin{array}{c} \text{Malignant}\;:\;q_{\text{1},n}=\frac{{\hat{q}}_{\text{1},n}-\text{mean}\left({\hat{q}}_{\text{1}}\right)}{\text{mean}\left({\hat{q}}_{\text{2}}\right)-\text{mean}\left({\hat{q}}_{\text{1}}\right)} \end{array}$$ (1)

$$\begin{array}{c} \text{Benign}\;:\;q_{\text{2},n}=\frac{{\hat{q}}_{\text{2},n}-\text{mean}\left({\hat{q}}_{\text{1}}\right)}{\text{mean}\left({\hat{q}}_{\text{2}}\right)-\text{mean}\left({\hat{q}}_{\text{1}}\right)}, \end{array}$$ (2)

where $q_{\text{1},n}$ and $q_{\text{2},n}$ are the normalized quantitative values. This normalization simplifies the analytical analysis since the mean of $q_{\text{1}}$ becomes zero and the mean of $q_{\text{2}}$ becomes one. Consequently, the number of variables that must be tracked in the analytical analysis is reduced from four (the means and standard deviations of $q_{\text{1}}$ and $q_{\text{2}}$) to two (the standard deviations of $q_{\text{1}}$ and $q_{\text{2}}$).

An overview of the variables used in this study is provided in Table 1.

**Table 1:** Overview and definitions of variables used in the analysis.

	Variable	Definition
1	$\hat{\mu };{\hat{\mu }}_{\text{1}}$; ${\hat{\mu }}_{\text{2}}$	Mean ADC value of study population; mean ADC of malignant lesions; mean ADC of benign lesions
2	$\hat{\sigma };\;{\hat{\sigma }}_{\text{1}}$; ${\hat{\sigma }}_{\text{2}}$	std(ADC) across study population; std(ADC) of malignant lesions; std(ADC) of benign lesions
3	N; $N_{\text{1}}$; $N_{\text{2}}$	sample size; group size of malignant lesions; group size of benign lesions; $N=N_{\text{1}}+N_{\text{2}}$
4	$\mu_{\text{1}};\;\mu_{\text{2}}$	Normalized versions of ${\hat{\mu }}_{\text{1}}$ and ${\hat{\mu }}_{\text{2}}$; $\mu_{\text{1}}=\text{0};\;\mu_{\text{2}}=\text{1}$
5	$\sigma_{\text{1}}$; $\sigma_{\text{2}}$	Normalized versions of ${\hat{\sigma }}_{\text{1}}$ and ${\hat{\sigma }}_{\text{2}}$
6	q	Normalized quantitative value (e.g., a single normalized ADC value)
7	CoV, $\overset{―}{CoV}$	Coefficient of variation; mean of CoV computed from the individual CoV values of the considered precision studies
8	$\sigma_{\text{CoV}}$	Normalized precision error of ADC values (derived from $\overset{―}{CoV}$)
9	std(AUC)	Finite N error: normalized std of AUC estimated by Monte Carlo and kernel density simulation
10	$q_{\text{1},\text{random}}$; $q_{\text{2},\text{random}}$	Randomized malignant and benign ADC values
11	$p_{\text{1}}(q);\;p_{\text{2}}(q)$	PDFs for malignant and benign cases
12	$q_{\text{error}}$	Measurement error
13	$p_{\text{precision}}\left(q_{\text{error}}\right)$	PDF of $q_{\text{error}}$
14	${\Delta AUC}_{\text{precision}}$	AUC reduction due to measurement imprecision: precision error

ADC = apparent diffusion coefficient; AUC = area under the curve; CoV = coefficient of variation; PDF = probability density function; std = standard deviation.

### Literature search for breast diffusion-weighted imaging studies

**Fig 2:**
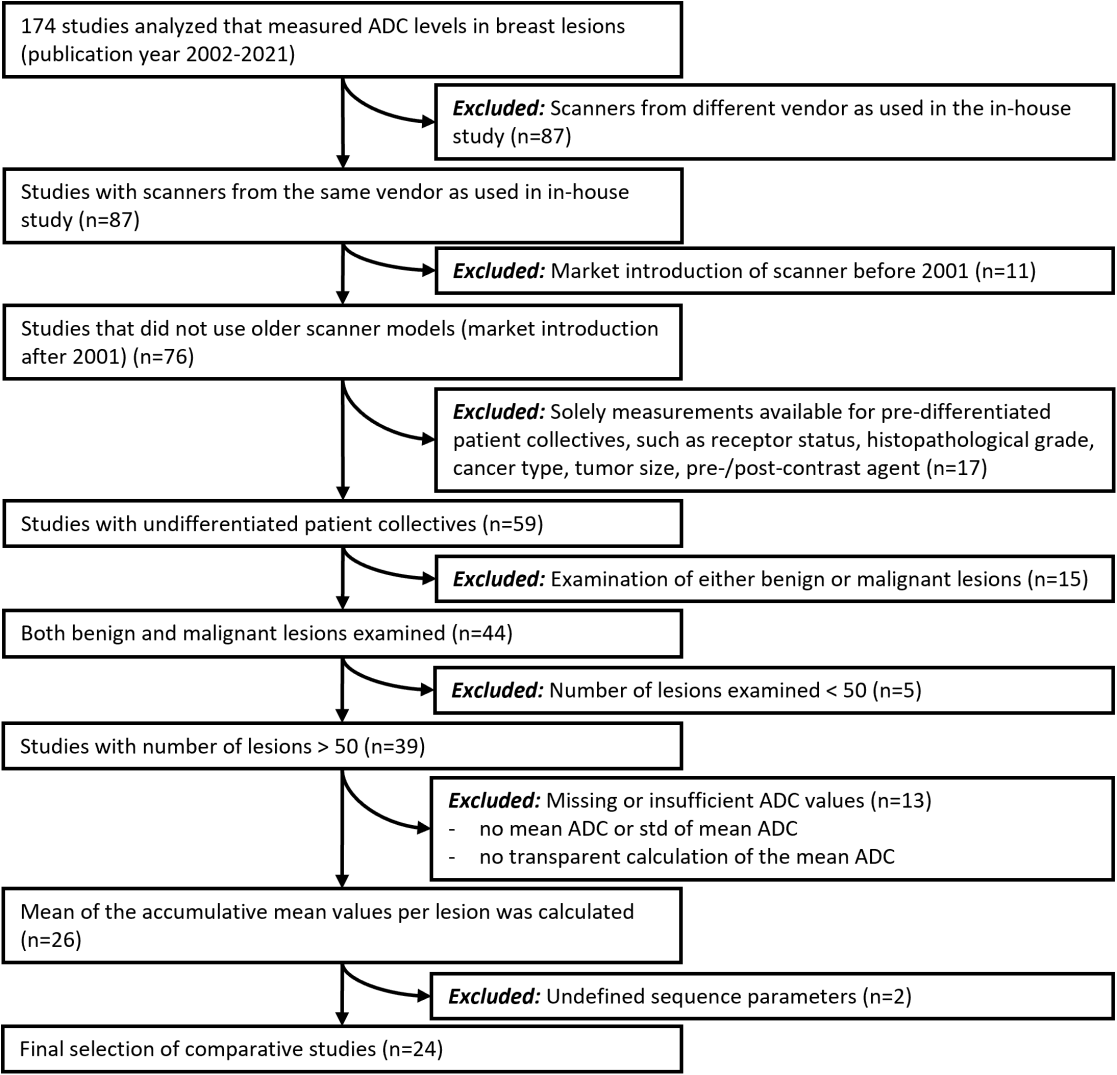
Flowchart of the literature selection process. From an initial 174 studies, 24 met the predefined inclusion criteria for quantitative ADC evaluation in breast DWI. Some inclusion criteria were necessary to make the included studies suitable for our analysis (e.g., data on *both* malignant and benign lesions was required, as well as the standard deviations of the measured ADCs). Other criteria were defined restrictively to ensure a high comparability of the included studies (e.g., due to the exclusion of studies performed with scanners from different vendors or of older scanners) and to ensure that high-quality studies were included (e.g., with sample sizes larger than 50). ADC = apparent diffusion coefficient; DWI = diffusion-weighted image; std = standard deviation.

The selected use case “DWI in breast MRI” was selected for its relevance and the availability of reported data. A literature research was performed using the PubMed database with the search term “Mamma AND DWI AND ADC” in December 2021. The retrospective search yielded n = 174 studies published between 2002 and 2021. From the resulting 174 studies (published between 2002 and 2021), 24 publications met the inclusion criteria (for further details please refer to Fig 2 and the supporting information (S1 File)). For each study, the reported mean μ and standard deviation σ of the ADCs of malignant and benign lesions were retrieved (malignant: ${\hat{\mu }}_{\text{1}}$ and ${\hat{\sigma }}_{\text{1}}$, benign: ${\hat{\mu }}_{\text{2}}$ and ${\hat{\sigma }}_{\text{2}}$), along with the group sizes of the malignant class ($N_{\text{1}}$) and the benign class ($N_{\text{2}}$).

Similarly as in the in-house study, the mean values of the two classes were normalized to 0 and 1, respectively ($\mu_{\text{1}}=\text{mean}\left(q_{\text{1}}\right)=\text{0}$ and $\mu_{\text{2}}=\text{mean}\left(q_{\text{2}}\right)=\text{1}$). The corresponding normalized standard deviations (malignant: $\sigma_{\text{1}}$and benign: $\sigma_{\text{2}}$) for both the in-house study and the reported studies were determined as follows:


$$\begin{array}{c} \sigma_{\text{1}}=\frac{{\hat{\sigma }}_{\text{1}}}{\left|{\hat{\mu }}_{\text{2}}-{\hat{\mu }}_{\text{1}}\right|} \end{array}$$ (3)

$$\begin{array}{c} \sigma_{\text{2}}=\frac{{\hat{\sigma }}_{\text{2}}}{\left|{\hat{\mu }}_{\text{2}}-{\hat{\mu }}_{\text{1}}\right|} \end{array}$$ (4)

Thus, one value of ${\hat{\mu }}_{\text{1}}$, ${\hat{\mu }}_{\text{1}}$, ${\hat{\sigma }}_{\text{1}}$, ${\hat{\sigma }}_{\text{2}}$, $\sigma_{\text{1}}$, and $\sigma_{\text{2}}$ was obtained for each of the included studies.

### Literature search for reported coefficients of variation

While reports on the accuracy of MRI are available [11,12], accuracy is usually more difficult to assess than precision. Therefore, we here focused on precision errors, which can be expressed by the coefficient of variation (CoV). A literature search was performed for reported CoV values in breast DWI using the Pubmed database and the search term “coefficient of variation AND “breast OR mamma” AND ADC AND DWI” in December 2021, with 10 studies included. From the 10 CoV values of the 10 studies, the mean, $\overset{―}{CoV}$, was computed (see further details, including inclusion criteria, in the supporting information (S1 File)).

The CoV was converted into a standard deviation $\sigma_{\text{CoV}}$. To obtain this standard deviation, the CoV was multiplied with the mean ADC value. One could treat benign and malignant lesions separately and obtain two standard deviations. However, for simplicity, the overall mean ADC value of all lesions including a conversion to the normalized space was calculated here as follows:


$$\begin{array}{c} \sigma_{\text{CoV}}\approx \frac{\text{mean}\left({\hat{\mu }}_{\text{1}},{\hat{\mu }}_{\text{2}}\right)\cdot \overset{―}{CoV}\;}{\left|{\hat{\mu }}_{\text{2}}-{\hat{\mu }}_{\text{1}}\right|} \end{array}$$ (5)

Thus, $\overset{―}{CoV}\;$ was kept fixed. However, $\sigma_{\text{CoV}}$ varied among the included breast DWI studies because their ${\hat{\mu }}_{\text{1}}$ and ${\hat{\mu }}_{\text{2}}$ values differed. For the in-house study, $\sigma_{\text{CoV}}$ was calculated with the same formula.

### Finite N error: Assessment with Monte-Carlo simulations and kernel density estimations

For the quantitative evaluation of both types of error, the AUC was used as a common measure of diagnostic discriminatory power. In the following steps, the strength with which it is influenced by sampling variability (finite N error) and how it is influenced by measurement imprecision (precision error) were analyzed. The standard error of the AUC, std(AUC), was estimated by means of Monte Carlo simulations performed in Matlab (Version 2022b, MathWorks, Natick, USA). In the AUC analysis, cutoff values were used that ranged from q=−2.5 to q=3. In this analysis, lesions with q-values smaller than the cutoff value were classified as malignant while the remaining lesions were classified as benign. The simulation was performed in normalized space for comparability and was carried out as described in the following.$\;N_{\text{1}}$ random numbers were generated for the malignant class and $N_{\text{2}}$ random numbers for the benign class.

These random numbers were drawn from Gaussian distributions using the normalized means ($\mu_{\text{1}}=\text{0}$ and $\mu_{\text{2}}=\text{1}$) and standard deviations ($\sigma_{\text{1}}$ and $\sigma_{\text{2}}$) of the respective study. In pseudo-code, the random numbers were generated as follows:


$$\begin{array}{c} \text{Malignant lesion}\;:\;q_{\text{1},n,\text{random}}=\text{randn}(\text{1})\cdot \sigma_{\text{1}} \end{array}$$ (6)

$$\begin{array}{c} \text{Benign lesion}\;:\;q_{\text{2},n,\text{random}}=\text{randn}(\text{1})\cdot \sigma_{\text{2}}+\text{1} \end{array}$$ (7)

Here, randn(1) is the Matlab function that generates a normally distributed random number with a mean 𝜇 = 0 and a standard deviation 𝜎 = 1.

In contrast to the analysis of the literature studies, where only summary statistics (mean and standard deviation) were available and therefore a normal distribution was assumed, all individual ADC values were available for the in-house study. This allowed for the underlying probability density functions (PDFs) of the malignant and benign lesions to be determined empirically using kernel density estimation rather than prescribing a specific distribution shape. The advantage of this approach is that the complete distribution structure of the data, including possible asymmetries or multi-peakedness, is preserved. This allowed for the class separation and the finite N error to be modeled more realistically. The “kernel” parameter controlled the degree of smoothing of the empirical density function by specifying the width of the Gaussian kernel used. For the in-house study, the random numbers were generated by picking a random normalized ADC value that had been obtained in the study ($q_{\text{1}}$ for the malignant and $q_{\text{2}}$ for the benign class) and adding a small normally distributed random variable. In pseudo-code:


$$\begin{array}{c} \text{Malignant lesion}\;:\;q_{\text{1},n,\text{random}}=q_{\text{1},\text{randi}\left(N_{\text{1}}\right)}+\text{randn}(\text{1})\cdot \sigma_{\text{kernel}} \end{array}$$ (8)

$$\begin{array}{c} \text{Benign lesion}\;:\;q_{\text{2},n,\text{random}}=q_{\text{2},\text{randi}\left(N_{\text{2}}\right)}+\text{randn}(\text{1})\cdot \sigma_{\text{kernel}} \end{array}$$ (9)

Here, randi(N) draws an integer from a uniform distribution between 1 and N. The value $\sigma_{\text{kernel}}$ was set to 0.1. The purpose of introducing the $\sigma_{\text{kernel}}$-term was to mimic a kernel density estimation of the true distribution from the available data points. The choice of $\sigma_{\text{kernel}}$ is described in detail in the subsection “Additional precision errors for the internal study”.

For both in-house study and literature studies, the AUCs were calculated from these random q-values. This process was repeated 10,000 times. From the thus obtained 10,000 AUC values (per study), the mean AUC and the standard error, std(AUC), were computed. Using this approach, std(AUC) quantified the variation of the AUC that originated from the random sampling of q-values. The precision error was not explicitly included in this calculation. However, it was contained implicitly because the published values of $\sigma_{\text{1}}$ and $\sigma_{\text{2}}$ were derived with an immanent precision error that contributes to the standard deviation of the q-values found in the studies. The thus obtained std(AUC) value was the finite N error.

**Fig 3:**
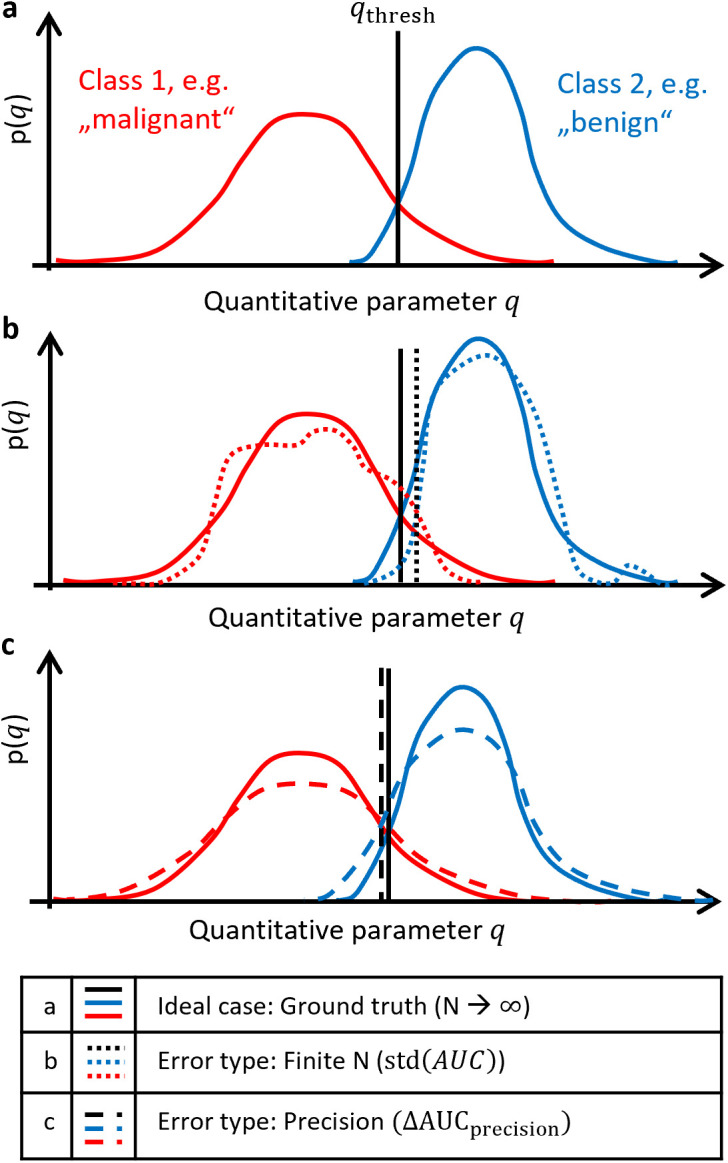
Illustration of error types affecting class separation. a) Ideal case with perfectly known PDFs for two classes. b) Finite N error leads to incorrectly estimated PDFs. Consequently, cutoff ${\text{q}}_{\text{thresh}}$ and AUC are randomly estimated, resulting in the standard error of AUC, std(AUC). **c)** Precision error broadens PDFs due to measurement noise, reducing AUC by $\Delta {\text{AUC}}_{\text{precision}}$. AUC = area under the receiver operating characteristic curve; N = sample size; PDF = probability density function.

Figs 3a and 3b visualize the finite N error, represented by the standard deviation of the AUC, which arises from random variability in the estimated PDFs due to limited sample size. Due to limited precision, one does not measure the true quantitative value q, where q is the normalized ADC in the case of DWI, but an imprecise estimate $q+q_{\text{error}}$. $q_{\text{error}}$ describes the difference between the ideal ADC (without measurement imprecision) and the ADC reduced by limited precision. The PDF of $q_{\text{error}}$ is $p_{\text{precision}}\left(q_{\text{error}}\right)$.

### Precision error: Analytical assessment using normal probability density functions

The precision error was assessed with the following theoretical consideration. Let the quantitative parameter be called q. Once again, q represents the ADC normalized such that the mean of classes 1 and 2 equal 0 and 1, respectively. The distribution of q for malignant and benign classes is described by the two PDFs $p_{\text{1}}(q)$ and $p_{\text{2}}(q)$, respectively. Here, $p_{\text{1}}(q)$ and $p_{\text{2}}(q)$ were modeled as normal functions N(q,mean, variance); see Fig 3a:


$$\begin{array}{c} p_{\text{1}}(q)=N\left(q,\;\text{0},\overset{\text{2}}{\underset{\text{1}}{\sigma }}\right) \end{array}$$ (10)

$$\begin{array}{c} p_{\text{2}}(q)=N\left(q,\;\text{1},\overset{\text{2}}{\underset{\text{2}}{\sigma }}\right) \end{array}$$ (11)

Here, $\overset{\text{2}}{\underset{\text{1}}{\sigma }}$ and $\overset{\text{2}}{\underset{\text{2}}{\sigma }}$ are the variances of the two normal distributions. The means of the distributions are equal to 0 and 1, respectively. The respective AUC is given below (see supporting information (S1 File)):


$$\begin{array}{c} AUC=\frac{\text{1}}{\text{2}}+\frac{\text{1}}{\text{2}}\text{erf}\left(\frac{\text{1}}{\sqrt{\text{2}\overset{\text{2}}{\underset{\text{1}}{\sigma }}+\text{2}\overset{\text{2}}{\underset{\text{2}}{\sigma }}}}\right), \end{array}$$ (12)

where erf  denotes the error function. This AUC value represents the ideal case of vanishing precision error and $N_{\text{1}}\to \infty$ and $N_{\text{2}}\to \infty$.

As visualized in Fig 3c, the PDFs are broadened by this error. This broadening can be described with a convolution:


$$\begin{array}{c} p_{\text{1},\text{precision}}(q)=p_{\text{1}}(q)*p_{\text{precision}}(q) \end{array}$$ (13)

$$\begin{array}{c} p_{\text{2},\text{precision}}(q)=p_{\text{2}}(q)*p_{\text{precision}}(q) \end{array}$$ (14)

While $\;p_{\text{precision}}\left(q_{\text{error}}\right)$ may have involved functional shapes, the simplification that it is Gaussian with mean = 0 and standard deviation $\sigma_{\text{CoV}}$ was made. That is, $\sigma_{\text{CoV}}$ is the standard deviation that one obtains when measuring the same dataset several times. For simplicity, $\sigma_{\text{CoV}}$ was assumed to be identical for both classes here. Thus,


$$\begin{array}{c} p_{\text{precision}}\left(q_{\text{error}}\right)=N\left(q_{\text{error}},\text{0},\overset{\text{2}}{\underset{\text{CoV}}{\sigma }}\right). \end{array}$$ (15)

The convolution of a Gaussian function with a Gaussian function is a Gaussian function. Hence, in the case of two Gaussian PDFs, this broadening leads to the following PDFs:


$$\begin{array}{c} p_{\text{1},\text{precision}}(q)\;=N\left(q,\text{0},\overset{\text{2}}{\underset{\text{1}}{\sigma }}+\overset{\text{2}}{\underset{\text{CoV}}{\sigma }}\right) \end{array}$$ (16)

$$\begin{array}{c} p_{\text{2},\text{precision}}(q)\;=\;N\left(q,\text{1},\overset{\text{2}}{\underset{\text{2}}{\sigma }}+\overset{\text{2}}{\underset{\text{CoV}}{\sigma }}\right) \end{array}$$ (17)

Accordingly, the variances $\overset{\text{2}}{\underset{\text{1}}{\sigma }}$ and $\overset{\text{2}}{\underset{\text{CoV}}{\sigma }}$ are additive, as are $\overset{\text{2}}{\underset{\text{2}}{\sigma }}$ and $\overset{\text{2}}{\underset{\text{CoV}}{\sigma }}$.

The corresponding AUC is as follows:


$$\begin{array}{c} {AUC}_{\text{precision}}=\frac{\text{1}}{\text{2}}+\frac{\text{1}}{\text{2}}\text{erf}\left(\frac{\text{1}}{\sqrt{\text{2}\overset{\text{2}}{\underset{\text{1}}{\sigma }}+\text{2}\overset{\text{2}}{\underset{\text{2}}{\sigma }}+\text{4}\overset{\text{2}}{\underset{\text{CoV}}{\sigma }}}}\right) \end{array}$$ (18)

This AUC value represents the case with limited precision ($\sigma_{\text{CoV}}>\text{0}$) and $N_{\text{1}}\to \infty$ and $N_{\text{2}}\to \infty$.

The drop in AUC due to limited precision is as follows:


$$\begin{array}{c} {\Delta AUC}_{\text{precision}}=AUC-{AUC}_{\text{precision}}. \end{array}$$ (19)

${\Delta AUC}_{\text{precision}}$ is the precision error.

### Additional precision error for the in-house study: Numerical assessment using probability density functions

**Fig 4:**
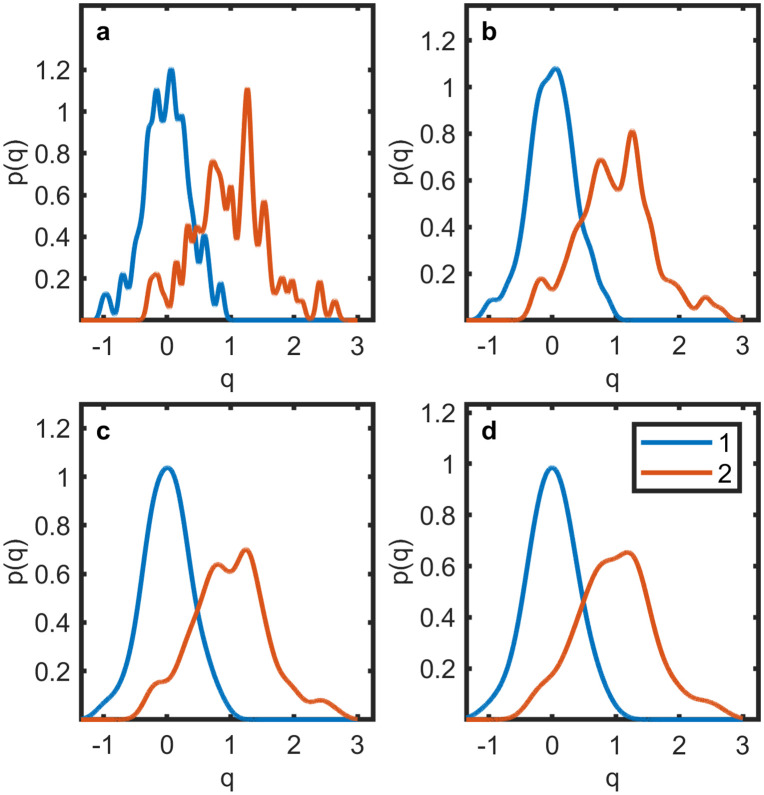
Estimated probability density functions for malignant and benign lesions at different kernel widths. The range of different kernel widths presented in Figs 4a–d is $\sigma_{\text{kernel}}$ = 0.05–0.2. **a)** $\sigma_{\text{kernel}}=$ 0.05, **b)** $\sigma_{\text{kernel}}=$ 0.1, **c)** $\sigma_{\text{kernel}}=$ 0.15, and **d)** $\sigma_{\text{kernel}}=$ 0.2. Each panel shows the effect of kernel size on the smoothness of the reconstructed distributions based on the in-house dataset. Red lines represent the malignant class, blue lines the benign class. p(q) = probability density function; q = normalized quantitative parameter.

For the in-house study, the $N_{\text{1}}$ data points of class 1 and $N_{\text{2}}$ data points of class 2 were called $q_{\text{1},n}$ and $q_{\text{2},n}$, respectively. In the absence of a precision error, the PDFs for the in-house study were estimated with a kernel density estimation:


$$\begin{array}{c} p_{\text{1}}(q)=\frac{\text{1}}{N_{\text{1}}}\sum_{n}^{N_{\text{1}}}N\left(q-q_{\text{1},n},\text{0},\overset{\text{2}}{\underset{\text{kernel}}{\sigma }}\right), \end{array}$$ (20)

$$\begin{array}{c} p_{\text{2}}(q)=\frac{\text{1}}{N_{\text{2}}}\sum_{n}^{N_{\text{2}}}N\left(q-q_{\text{2},n},\text{0},\overset{\text{2}}{\underset{\text{kernel}}{\sigma }}\right), \end{array}$$ (21)

where $N\left(q-q_{i};\text{0},\overset{\text{2}}{\underset{\text{kernel}}{\sigma }}\right)$ is a normal function with zero mean and standard deviation $\sigma_{\text{kernel}}$. Fig 4 shows the reconstructed PDFs for $\sigma_{\text{kernel}}=$ 0.05, 0.1, 0.15, and 0.2. In the subsequent analysis, $\sigma_{\text{kernel}}=\text{0}.\text{1}$ was used. This value was considered a good compromise between retaining detail and reducing spurious peaks from sampling noise. One the one hand, it smeared out most peaks visible with $\sigma_{\text{kernel}}=\text{0}.\text{0}\text{5}$, which were assumed to be sampling artifacts. On the other hand, $\sigma_{\text{kernel}}$ was kept as small as possible to minimize the blurring of the PDFs that accompanies larger $\sigma_{\text{kernel}}$ values.

For the case with measurement imprecision, that is, for $\sigma_{\text{CoV}}>\text{0}$, the approach used for the literature studies was adopted to estimate the PDFs as follows:


$$\begin{array}{c} p_{\text{1},\text{precision}}\left(q,\sigma_{\text{CoV}}\right)=\frac{\text{1}}{N_{\text{1}}}\sum_{n}^{N_{\text{1}}}N\left(q-q_{\text{1},n};\text{0},\overset{\text{2}}{\underset{\text{kernel}}{\sigma }}+\overset{\text{2}}{\underset{\text{CoV}}{\sigma }}\right), \end{array}$$ (22)

$$\begin{array}{c} p_{\text{2},\text{precision}}\left(q,\sigma_{\text{CoV}}\right)=\frac{\text{1}}{N_{\text{2}}}\sum_{n}^{N_{\text{2}}}N\left(q-q_{\text{2},n};\text{0},\overset{\text{2}}{\underset{\text{kernel}}{\sigma }}+\overset{\text{2}}{\underset{\text{CoV}}{\sigma }}\right). \end{array}$$ (23)

Then, the area under the curve was computed by numerically computing these PDFs at 5000 points $q_{n}$ ranging from −2.5 to 3 and integrating numerically using the following equations. Different numbers of points were tested in preparatory evaluations, and 5000 points were found to be sufficient to ensure convergence of the numerical result.


$$\begin{array}{c} \text{Sensitivity}\left(q_{n},\sigma_{\text{CoV}}\right)=\sum_{m=\text{1}}^{n}p_{\text{1},\text{precision}}\left(q_{m},\sigma_{\text{CoV}}\right)\cdot \left(q_{m+\text{1}}-q_{m}\right) \end{array}$$ (24)

$$\begin{array}{c} \text{Specificity}\left(q_{n},\sigma_{\text{CoV}}\right)=\text{1}-\sum_{m=\text{1}}^{n}p_{\text{2},\text{precision}}\left(q_{m},\sigma_{\text{CoV}}\right)\cdot \left(q_{m+\text{1}}-q_{m}\right). \end{array}$$ (25)

$$\begin{array}{c} {AUC}_{\text{precision}}\left(\sigma_{\text{CoV}}\right)=\sum_{n=\text{1}}^{\text{5000}}\text{Sensitivity}\left(q_{n},\sigma_{\text{CoV}}\right)\cdot \left(\text{Specificity}\left(q_{n},\sigma_{\text{CoV}}\right)-\text{Specificity}\left(q_{n}+\text{1},\sigma_{\text{CoV}}\right)\right) \end{array}$$ (26)

Then, the drop in AUC due to $\sigma_{\text{CoV}}$ was computed with the formula


$$\begin{array}{c} {\Delta AUC}_{\text{precision}}={AUC}_{\text{precision}}(\text{0})-{AUC}_{\text{precision}}\left(\sigma_{\text{CoV}}\right). \end{array}$$ (27)

### Sample size needed for equivalence of errors

The AUC’s standard error, std(AUC), scales as follows:


$$\begin{array}{c} \text{std}(AUC)\propto \frac{\text{1}}{\sqrt{N}} \end{array}$$ (28)

to a good approximation, where $N=N_{\text{1}}+N_{\text{2}}$. If $\text{std}(AUC)=\;\;/\;{\Delta AUC}_{\text{precision}}$ for the particular N value of a particular study, one obtains an equivalence of errors by increasing (or decreasing) the sample size:


$$\begin{array}{c} N_{\text{equality of errors}}=N\cdot {\left(\frac{\text{std}(AUC)}{{\Delta AUC}_{\text{precision}}}\right)}^{\text{2}}. \end{array}$$ (29)

$N_{\text{equality of errors}}$ was computed for all previous studies and the in-house study using the mean coefficient of variation $\overset{―}{CoV}\;$, which had been obtained from the literature search.

#### Dependence of the sample size needed for equivalence of errors on the coefficient of variation.

$N_{\text{equality of errors}}$ was additionally computed for the following CoV values: 1%, 2%, 3%, 5%, 7%, 10%, 13%, 16%, and 20% (also with 10,000 repetitions each). The mutual dependency was then evaluated with the fit of a power law relation:


$$\begin{array}{c} N_{\text{equality of errors}}=a\cdot CoV^{b}. \end{array}$$ (30)

For the fit, the median $N_{\text{equality of errors}}$ among all studies was used, and Eq. 31 was linearized: $\text{log}N_{\text{equality of errors}}=\text{log}a+b\cdot \text{log}CoV$. Then, loga and b were fitted with a Levenberg–Marquardt fit.

Theoretically, one would expect the following relationship. For a small $\sigma_{\text{CoV}}$ (or small CoV values), ${\Delta AUC}_{\text{precision}}$ may be approximated as follows (see supporting information (S1 File)):


$$\begin{array}{c} {\Delta AUC}_{\text{precision}}\approx \frac{\text{exp}\left(-\frac{\text{1}}{\text{2}\overset{\text{2}}{\underset{\text{1}}{\sigma }}+\text{2}\overset{\text{2}}{\underset{\text{2}}{\sigma }}}\right)\cdot \overset{\text{2}}{\underset{\text{CoV}}{\sigma }}}{\sqrt{\text{2}\pi }\cdot {\left(\overset{\text{2}}{\underset{\text{1}}{\sigma }}+\overset{\text{2}}{\underset{\text{2}}{\sigma }}\right)}^{\frac{\text{3}}{\text{2}}}}. \end{array}$$ (31)

Thus, ${\Delta AUC}_{\text{precision}}$ scales like ${\Delta AUC}_{\text{precision}}\propto \overset{\text{2}}{\underset{\text{CoV}}{\sigma }}\propto {CoV}^{\text{2}}$, and $N_{\text{equality of errors}}$ scales like (see Eq. 29)


$$\begin{array}{c} N_{\text{equality of errors}}\propto \overset{-\text{2}}{\underset{\text{precision}}{\Delta AUC}}\propto \overset{-\text{4}}{\underset{\text{CoV}}{\sigma }}\propto {CoV}^{-\text{4}}. \end{array}$$ (32)

### Availability of code

The programming MATLAB code for the described analyses is provided as supporting information (S2 File) as well as a MATLAB code to perform your own analysis (S3 File). Additional files to run both Matlab codes are provided (S4 File, S5 File, S6 File).

## Results

### Apparent diffusion coefficient values in benign and malignant breast lesions

#### In-house-study.

In total, 171 lesions were included in our in-house study. The mean ADCs per class (0.79 µm²/ms for malignant lesions and 1.32 µm²/ms for benign lesions) and their respective standard deviations (0.20 µm²/ms and 0.32 µm²/ms) are given in Table 2. The demographic characteristics, selection process, histopathological classification, and imaging protocol are detailed in the supporting material (S1 File). Table 3 shows the normalized in-house study values of $\mu_{\text{1}}$, $\mu_{\text{2}}$, $\sigma_{\text{1}}$, and $\sigma_{\text{2}}$. The values of $\mu_{\text{1}}$ and $\mu_{\text{2}}$ just equal zero and one, respectively, due to the chosen normalization.

**Table 2:** Mean and standard deviation of ADC values for malignant and benign breast lesions in the in-house study and 24 published studies.

Study	N	$N_{1}$ malignant	$N_{2}$ benign	${\hat{\mu }}_{1}$ mean ADC malignant (µm²/ms)	${\hat{\sigma }}_{1}$ std ADC malignant (µm²/ms)	${\hat{\mu }}_{1}$ mean ADC benign (µm²/ms)	${\hat{\sigma }}_{2}$ std ADC benign (µm²/ms)
In-house	171	88	83	0.79	0.20	1.32	0.32
1 [13]	131	66	65	0.87	0.13	1.64	0.47
2 [14]	246	146	100	0.96	0.19	1.32	0.22
3 [15]	72	46	26	0.90	0.15*	1.86	0.44*
4 [16]	213	143	70	0.84	0.28	1.42	0.31
5 [17]	116	72	44	1.12	0.24	1.26	0.29
6 [18]	210	136	74	0.90	0.24	1.43	0.37
7 [19]	89	68	21	0.84	0.16*	1.38	0.49*
8 [20]	144	112	32	0.88	0.19	1.14	0.23
9 [21]	95	48	47	1.14	0.19	1.49	0.25
10 [22]	80	58	22	1.20	0.22	1.99	0.21*
11 [23]	61	34	27	1.00	0.18	1.66	0.23
12 [24]	56	34	22	0.95	0.29	1.52	0.33
13 [25]	85	39	46	1.03	0.19	1.68	0.27
14 [26]	181	89	92	0.83	0.19	1.41	0.24*
15 [27]	104	20	84	1.06	0.27	1.53	0.38
16 [28]	326	259	67	1.02	0.17	1.57	0.26
17 [29]	170	85	85	1.11	0.33	1.81	0.46
18 [30]	98	57	41	1.02	0.18	1.48	0.33
19 [31]	72	38	34	1.06	0.20	1.69	0.23
20 [32]	169	106	63	1.04	0.29	1.42	0.46
21 [33]	115	88	27	0.89	0.28	1.10	0.34
22 [34]	106	91	15	0.98	0.19	1.50	0.20
23 [35]	61	52	9	0.98	0.24	1.51	0.26
24 [36]	111	63	48	0.73	0.24	1.19	0.42
**Mean of reported studies**	129	80	49	0.97	0.22	1.50	0.32

ADC = apparent diffusion coefficient; $\hat{\mu }$ = mean value; $\hat{\sigma }$ = std = standard deviation.

* = adapted values (see the supporting information for individual details (S1 File)).

**Table 3:** Normalized mean and standard deviation of ADC values, and $\sigma_{𝐂𝐨𝐕}$ for malignant and benign lesions in the in-house and reported studies.

Study	$\mu_{1}$ mean ADC malignant (µm²/ms)	$\sigma_{1}$ std ADC malignant (µm²/ms)	$\mu_{2}$ mean ADC benign (µm²/ms)	$\sigma_{2}$ std ADC benign (µm²/ms)	$\sigma_{CoV}$ normalized precision error
In-house	0	0.37	1	0.60	0.40
1 [13]	0	0.17	1	0.61	0.32
2 [14]	0	0.53	1	0.61	0.63
3 [15]	0	0.16	1	0.46	0.29
4 [16]	0	0.48	1	0.53	0.39
5 [17]	0	1.71	1	2.07	1.70
6 [18]	0	0.45	1	0.70	0.44
7 [19]	0	0.30	1	0.91	0.41
8 [20]	0	0.73	1	0.88	0.78
9 [21]	0	0.54	1	0.71	0.75
10 [22]	0	0.28	1	0.27	0.43
11 [23]	0	0.27	1	0.35	0.40
12 [24]	0	0.51	1	0.58	0.43
13 [25]	0	0.29	1	0.42	0.42
14 [26]	0	0.33	1	0.41	0.39
15 [27]	0	0.57	1	0.81	0.55
16 [28]	0	0.31	1	0.47	0.47
17 [29]	0	0.47	1	0.66	0.42
18 [30]	0	0.39	1	0.72	0.54
19 [31]	0	0.32	1	0.37	0.44
20 [32]	0	0.76	1	1.21	0.65
21 [33]	0	1.33	1	1.62	0.95
22 [34]	0	0.37	1	0.38	0.48
23 [35]	0	0.45	1	0.49	0.47
24 [36]	0	0.52	1	0.91	0.42
**Mean of reported studies**	0	0.51	1	0.71	0.54

ADC = apparent diffusion coefficient; μ = mean value; σ = std = standard deviation; $\sigma_{\text{CoV}}$ = normalized precision error of ADC values (derived from $\overset{―}{CoV}$).

#### Literature search.

The literature search yielded n = 24 studies. The retrieved means and standard deviations are summarized in Table 2. The total sample size $N=N_{\text{1}}+N_{\text{2}}$ ranged from 41 to 326, with a mean value of 129. The ADC values of malignant lesions ranged from 0.79 µm²/ms to 1.20 µm²/ms, with a mean value of 0.97 µm²/ms. The ADC values of benign lesions were higher in each study and ranged from 1.10 µm²/ms to 1.99 µm²/ms, with a mean value of 1.50 µm²/ms.

The mean $\sigma_{\text{1}}$ value of the included studies was 0.51, and the mean $\sigma_{\text{2}}$ value was 0.71 (see Table 3). For example, study 10 [22] stood out somewhat with rather small normalized standard deviations ($\sigma_{\text{1}}=$ 0.28 and $\sigma_{\text{2}}=\;$0.27). Studies 5 [17] and 21 [33] had the largest $\sigma_{\text{1}}$ and $\sigma_{\text{2}}$ values among the considered studies (study 5: $\sigma_{\text{1}}=\;$1.71 and $\sigma_{\text{2}}=\;$2.07; study 21: $\sigma_{\text{1}}=\;$1.33 and $\sigma_{\text{2}}=\;$1.62).

### Literature search: Reported coefficients of variation

Table 4 summarizes the 10 considered studies on the CoV in breast DWI. The actual study design varied between the studies regarding the segmentation procedure, the retest approach, and the considered tissue. The mean CoV across all 10 studies, $\overset{―}{CoV}$, was 7.7% ± 3.9%. The $\sigma_{\text{CoV}}$ values for the 24 studies derived with this $\overset{―}{CoV}$ value are stated in Table 3. They ranged from 0.11 (for study 3 [15]) to 0.65 (for study 5 [17]). The mean $\sigma_{\text{CoV}}$ value was 0.21.

**Table 4:** Reported test–retest coefficients of variation (CoV) for mean ADC values in benign and malignant breast lesions.

Author	Year	Study type	N patients	Scanner type	Segmentation procedure	𝐂𝐨𝐕 of ADC [%]	Definition of 𝐂𝐨𝐕	Method	Dignity of lesion/tissue
Jerome et al. [37]	2021	Prospective	21	3 T, Siemens	3D VOI, manually drawn on the DWI	9.41	Voxel-wise repeat-measures CoV	2nd scan after 7 days	Benign
Newitt et al. [38]	2020	Retrospective	71	1.5–3 T, any manufacturer	3D ROI, manually drawn on the DWI	5.36	wCV = mean [variance (test, retest)/mean2 (test, retest)] (1/2)	Repositioned	Malignant
Newitt et al. [39]	2020	Prospective	71	All, any manufacturer	3D ROI, manually drawn on the DWI	4.8	wCV (unit-less) = 100% * wSD/mean	Repositioned	Malignant
				1.5 T		4.7			
				3 T		5.1			
Almeida et al. [40]	2017	Retrospective	76	1.5 T, GE Healthcare	2 ROIs, manually drawn	7.03	Overall CoV	No further information available	Benign and malignant
Spick et al. [41]	2016	Prospective	40	3 T, Siemens	2D ROIs, darkest part of the lesion on the ADC map	3.2-8.3	Not available	Two scans on consecutive days	Benign and malignant
Aliu et al. [42]	2014	Prospective	9	1.5 T, GE Healthcare	ROI manually drawn on the T2w images	18; 20	bCV = 100 * SD/mean	Two scans within two consecutive months	FGT
						11	wCV = 100 * wSD/overall mean		
Mürtz et al. [43]	2014	Prospective	25	1.5 T, Philips Healthcare	2D ROI, manually drawn on the DWI	2.6-6.3	CV = SD/mean	Repeated with modified volume	Benign and malignant
Tagliafico et al. [44]	2012	Prospective	60	3 T, GE Healthcare	3 ROIs per patient, manually drawn on the DTI-derived map	15 (DTI)	Within-patient CoV, no further details available	Repetition of reading after 4 weeks	FGT
Partridge et al. [45]	2010	Prospective	12	1.5 T, GE Healthcare	2D ROIs, defined square of pixels	4.5 (DTI)	wCV, no further details available	Repositioned	FGT
Partridge et al. [46]	2001	Prospective	8	1.5 T, GE Medical Systems	2D ROI on the ADC map	5.5	CV = SD/mean	One scan per week for 4 weeks	FGT

bCV = between-subject coefficient of variation; CoV = coefficient of variation; DWI = diffusion-weighted image; FGT = fibroglandular tissue; ROI = region of interest; wCV = within-subject coefficient of variation; VOI = volume of interest; wSD = within-subject standard deviation.

### Finite N error: Results from Monte-Carlo- and kernel-size-simulations and PDF analysis

Table 5 summarizes the results from the Monte-Carlo simulations and the PDF-based analysis. The AUC values obtained with the Monte-Carlo simulation closely matched those obtained from the PDF analysis. The mean Monte-Carlo-simulation-derived AUC was 0.892, whereas the mean PDF-derived AUC was 0.891. The minimal AUC values were obtained for study 5 [17] (AUC = 0.646) and study 21 [33] (AUC = 0.683). The maximal AUC was obtained for study 10 [22] (AUC = 0.995).

**Table 5:** Results of Monte Carlo simulations and probability density function (PDF)–based error assessments.

Study	$\sigma_{CoV}$	𝐀𝐔𝐂 MC	𝐬𝐭𝐝(AUC) (%) MC	𝐀𝐔𝐂 PDF	${𝐀𝐔𝐂}_{𝐩𝐫𝐞𝐜𝐢𝐬𝐢𝐨𝐧}$ PDF	${\Delta AUC}_{𝐩𝐫𝐞𝐜𝐢𝐬𝐢𝐨𝐧}$ (%) PDF	${𝐍}_{𝐞𝐪𝐮𝐚𝐥𝐢𝐭𝐲\;𝐨𝐟\;𝐞𝐫𝐫𝐨𝐫𝐬}$
In-house (KD)	0.15	0.920	2.14	0.908	0.898	0.94	875
In-house (Gauss)	0.15	0.924	2.11	0.924	0.914	0.94	868
1 [13]	0.13	0.943	2.44	0.943	0.936	0.70	1,572
2 [14]	0.24	0.892	2.08	0.892	0.873	1.96	278
3 [15]	0.11	0.981	2.06	0.981	0.975	0.53	1,113
4 [16]	0.15	0.918	2.03	0.917	0.909	0.90	1,094
5 [17]	0.65	0.646	5.45	0.645	0.637	0.76	6,005
6 [18]	0.17	0.885	2.63	0.885	0.876	0.93	1,666
7 [19]	0.16	0.853	6.47	0.853	0.846	0.65	8,904
8 [20]	0.30	0.808	4.50	0.808	0.793	1.50	1,293
9 [21]	0.29	0.868	3.70	0.867	0.845	2.28	252
10 [22]	0.16	0.995	0.52	0.995	0.988	0.74	41
11 [23]	0.16	0.988	1.08	0.988	0.979	0.95	79
12 [24]	0.17	0.903	4.14	0.903	0.893	1.01	932
13 [25]	0.16	0.976	1.41	0.976	0.964	1.16	134
14 [26]	0.15	0.971	1.08	0.971	0.961	1.01	205
15 [27]	0.21	0.844	4.22	0.843	0.833	1.06	1,654
16 [28]	0.18	0.962	1.42	0.962	0.947	1.51	289
17 [29]	0.16	0.892	2.49	0.892	0.883	0.88	1,365
18 [30]	0.21	0.890	3.67	0.889	0.875	1.44	634
19 [31]	0.17	0.981	1.30	0.981	0.968	1.24	79
20 [32]	0.25	0.758	4.08	0.758	0.751	0.64	6,895
21 [33]	0.36	0.683	6.16	0.683	0.678	0.49	17,956
22 [34]	0.18	0.970	2.07	0.970	0.955	1.55	189
23 [35]	0.18	0.933	3.90	0.933	0.919	1.39	325
24 [36]	0.16	0.830	4.15	0.829	0.824	0.55	6,312
Mean	0.21	0.892	3.01	0.891	0.880	1.07	2,506
Median	0.17	0.903	2.49	0.903	0.893	0.95	932

AUC = area under the curve; std(AUC) = finite N error; ${\Delta AUC}_{\text{precision}}$ = precision error; $\sigma_{\text{CoV}}$ = normalized precision error (derived from ($\overset{―}{CoV}$); MC = Monte-Carlo Simulation; PDF = probability density function; std = standard deviation. “In-house (KD)”: This analysis was performed by modelling the PDFs with the kernel density estimation approach. “In-house (Gauss)”: This analysis was performed in the same way as for the literature studies by modelling the PDFs with normal functions that had the in-house data means and standard deviations. For the calculation of the mean and median (last two lines), “In-house (Gauss)” was used.

The mean value of std(AUC) was 3.01%. Generally, a negative correlation was seen between std(AUC) and AUC: higher std(AUC) tended to go with lower AUC. The minimal std(AUC) was obtained for study 10 (std(AUC) = 0.52%). The maximal std(AUC) values were obtained for study 21 (std(AUC) = 6.16%) and study 7 [19] (std(AUC) = 6.47%).

For the in-house study, both the results for normal PDFs and the kernel density derived PDFs are stated in Table 5. The difference between the two approaches is small (e.g., $N_{\text{equality of errors}}=\;$875 vs 868).

### Precision error: Results of the probability density function analysis

Table 5 also summarizes the ${\Delta AUC}_{\text{precision}}$ values obtained from the PDF analysis. The mean ${\Delta AUC}_{\text{precision}}$ value was 1.00%. The minimal ${\Delta AUC}_{\text{precision}}$ was obtained for study 21 [33] (${\Delta AUC}_{\text{precision}}$ = 0.49%). The maximal ${\Delta AUC}_{\text{precision}}$ was obtained for study 9 [21] (${\Delta AUC}_{\text{precision}}\;$= 2.28%).

### Comparison of precision and finite N errors

**Fig 5:**
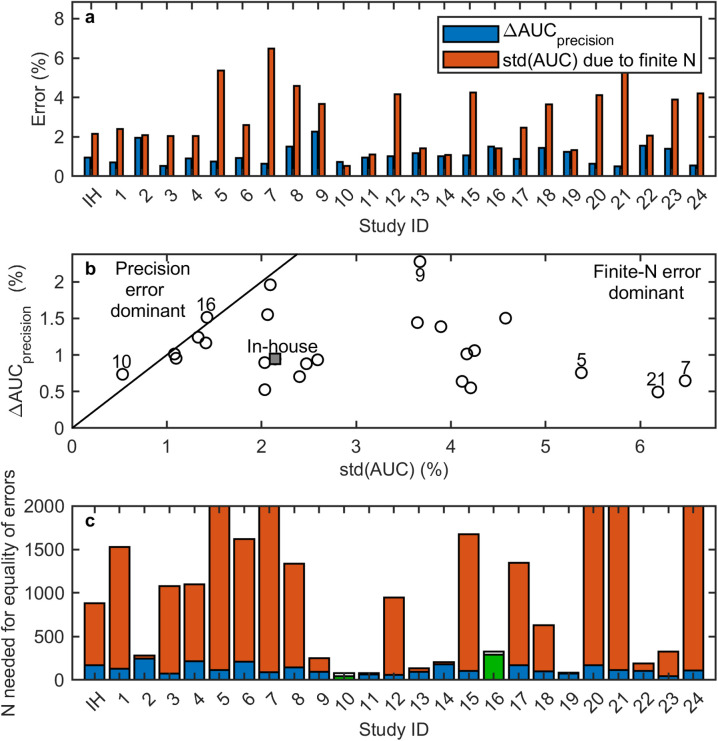
Comparison of finite N and precision errors across all studies. **a)** Bar plot of both error types, ${\Delta AUC}_{\text{precision}}$ and std(AUC). IH-KD = In-house study with kernel density PDF. IH-G = In-house study with Gaussian PDF approach. **b)** Respective scatter plot showing their relationship. Selected studies are labeled to illustrate representative positions across the range of finite N and precision errors, including our in-house study, outliers, and studies near the error-equivalence line. Square = IH-KD. Diamond = IH-G. **c)** Required sample size ${\text{N}}_{\text{equality of errors}}$ for equal contribution of both errors is plotted. ${\Delta AUC}_{\text{precision}}=\text{std}(AUC)$, assuming a scaling of $\text{std}(AUC)\propto (N{)}^{-\text{0}.\text{5}}$. AUC = area under the curve; IH = in-house; std = standard deviation; std(AUC) = error due to finite N; ${\Delta AUC}_{\text{precision}}$ = error due to imprecision.

Fig 5a shows ${\Delta AUC}_{\text{precision}}$ and std(AUC) for the published studies and the in-house study as a bar plot.

Fig 5b shows a scatter plot of the two errors. The finite N error is dominant (i.e., ${\Delta AUC}_{\text{precision}}<\text{std}(AUC)$) for all studies except studies 10 [22] and 16 [28]. Note that study 16 had the largest N. Study 10 had the lowest $\sigma_{\text{1}}$ and $\sigma_{\text{2}}$ values that originated from the rather small standard deviations ${\hat{\sigma }}_{\text{1}}$ and ${\hat{\sigma }}_{\text{2}},$ giving rise to a very high AUC. In this case, the reported class separation was extremely good, so the variation in AUC due to the finite sample size became very small.

For the in-house study, std(AUC)= 2.14% and ${\Delta AUC}_{\text{precision}}=\;$0.94% with the kernel density PDF approach and std(AUC)= 2.11% and ${\Delta AUC}_{\text{precision}}=\;$0.94% with the normal PDF approach. Due to the similarity of these values, the two data points lie closely together in Fig 5b (the gray-filled square and diamond). For some studies, ${\Delta AUC}_{\text{precision}}$ came close to std(AUC). For other studies, ${\Delta AUC}_{\text{precision}}\ll \text{std}(AUC)$. For example, study 7 [19] stood out, with a very large ratio between std(AUC)= 6.47% and ${\Delta AUC}_{\text{precision}}=\;$0.65%.

Fig 5c shows the sample size $N_{\text{equality of errors}}$ needed for an equality of the two errors. The bars have different colors: whenever $N_{\text{equality of errors}}\geq N$, the blue part represents N. The orange part represents $N_{\text{equality of errors}}-N$. Thus, the total height of the bars represents $N_{\text{equality of errors}}$. Whenever $N_{\text{equality of errors}}<N$, the green part represents $N_{\text{equality of errors}}$ and the white part represents $N-N_{\text{equality of errors}}$. Thus, the total height of the bars represents N.

The y-axis is cut at $N_{\text{equality of errors}}=\;$2000, since some studies exhibited very large $N_{\text{equality of errors}}$. For example, study 21 [33] stood out again, with $N_{\text{equality of errors}}\approx$ 18,000. The median of $N_{\text{equality of errors}}$ among all studies was 932.

### Dependence of the sample sized needed for equivalence of errors on the coefficient of variation

**Fig 6:**
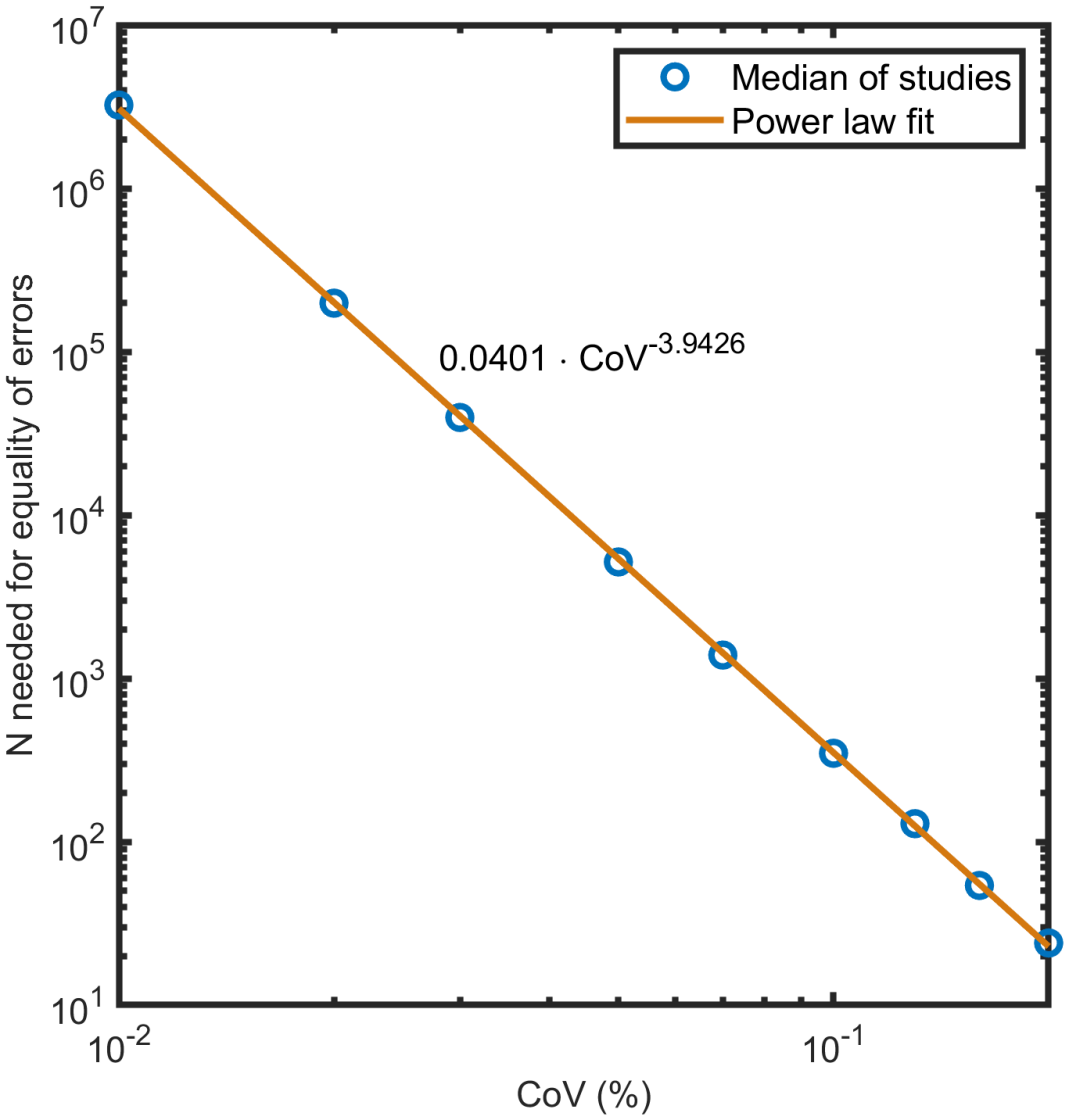
Dependency of the required sample size for error equivalence on the coefficient of variation. The median of ${\text{N}}_{\text{equality of errors}}$ among all studies for several CoV values (%) was fit by a power law fit. The curve shows that small changes in CoV lead to large increases in the sample size required to balance finite N and precision errors. CoV = coefficient of variation.

Fig 6 shows the dependency of $N_{\text{equality of errors}}$ on the CoV. The fit is in agreement with the $CoV^{-\text{4}}$ dependency predicted by Eq. 33. This illustrates that even small improvements in measurement precision (i.e., lower CoV) can lead to a disproportionately large reduction in the required sample size.

## Discussion

In this study, we investigated the size of two common error types in DWI ADC-based assessments of breast lesions: finite N errors represented by the standard error std(AUC) of the AUC and precision errors represented by ${\Delta AUC}_{\text{precision}}$. For the in-house study and the 24 considered published studies, we generally found $\text{std}(AUC)>{\Delta AUC}_{\text{precision}}$ with two exceptions (studies 7 [19] and 16 [28]). The median sample size of the considered studies was 109. The median sample size for which $\text{std}(AUC)={\Delta AUC}_{\text{precision}}$ was found to be 887 under the assumption that std(AUC) scales like $\text{std}(AUC)\propto N^{-\text{0}.\text{5}}$ (Eq. 28).

In this scenario of a dominant finite N error, the preferred action would thus generally be to increase N, if possible. In practice, the preferred action will depend on a variety of factors. Naturally, one would strive to minimize all sources of error as much as possible. However, a certain amount of error reduction may be associated with varying costs. Here, “cost” subsumes not only monetary costs but also other factors such as the burden that patients must face, for example, due to an increased scan time. Considering the sample size, the costs will usually increase linearly with N. Unfortunately, the associated standard deviation decreases only with $\text{1}/\sqrt{N}$ (see Eq. 28). Thus, the cost of improving std(AUC) will generally scale like $N^{\text{2}}$, which can quickly become insurmountable. In addition, going beyond a certain N becomes increasingly ineffective. For example, using N = 1000 for our in-house study would create a situation where the finite N error is no longer dominant. Then, investing time and effort in increasing the sample size would potentially not be as useful as spending resources to improve precision. For example, if resources are available, one could use a scanner with a higher field strength that provides higher Signal-to-Noise ratio [47], employ a receiver coil with more channels [48], use field probes to improve the image quality [49], invest in more elaborate, computationally demanding sequence and image reconstruction approaches [50,51], or allow more readers to evaluate the data. The most desirable action will depend heavily on the circumstances (e.g., availability of scanners, computation power, or availability of readers). Moreover, retrospective analyses of data available in a database, as in our in-house study, will naturally be assessed differently than prospective studies that involve the acquisition of new data. Generally, it will become increasingly difficult to reduce a certain error type, for example, the CoV; thus, a certain level of error may have to be accepted [38,52,53].

An evaluation such as that shown in Figs 5 and 6 can nonetheless help to guide one’s decisions and may be useful under different circumstances. For example, in the preparation phase of a prospective study, one usually performs sample size estimation and considers the effect size, the estimated measurement variability, the desired statistical power, the significance criterion, and the intended type of analysis (e.g., one- or two-tailed) [52,54–60]. Such an analysis could be supported by a joint consideration of the other errors to sharpen the judgment. For example, if one is not in the finite N error-dominated space (e.g., above the lines in Fig 5b), one might argue for reducing the sample size (which may have been inflated due to unrealistic expectations applied to its determination).

Similar to traditional power analyses, an evaluation such as that shown in Figs 5 and 6 could be used for sample size planning. This would include the following steps. First, retrieve the means and standard deviations of the two classes under consideration (e.g., benign and malignant) from the literature or from a pilot study and use them to compute the normalized standard deviations $\sigma_{\text{1}}$ and $\sigma_{\text{2}}$ (Eqs. 3 and 4). Second, obtain an estimate of the coefficient of variation from the literature or a pilot study and normalize it (see Eq. 5). Third, assume normality of the PDFs so that Eqs. 12 and 18 can be used to compute the decrease in AUC, ${\Delta AUC}_{\text{precision}}$ (Eq. 19), that arises from limited precision. Our finding that Gaussian PDFs yielded essentially the same results as kernel-density-based PDFs (square and diamond markers in Fig 5b) supports the general use of Gaussian PDFs, although further research may be warranted. Fourth, run a Monte Carlo simulation (Eqs. 6 and 7) to determine the standard error of the AUC, std(AUC), for the anticipated sample size, or for a range of anticipated sample sizes. Fifth, compare ${\Delta AUC}_{\text{precision}}$ and std(AUC) to determine the relative magnitude of these two errors and whether the precision error or the finite-N error is dominant. If the aim is to perform sample size planning, one potential approach is to set the anticipated sample size to $N_{\text{equality of errors}}$ (Eq. 29). Such a pre-study analysis might help to avoid an insufficient sample size. We provide MATLAB code for such an analysis (see S2 File).

In a post hoc analysis, it might be worthwhile to make a judgment based on the sizes of various errors (finite N, imprecision) to better estimate the trustworthiness of the obtained results. If the finite N error is dominant, researchers might mention this as a relevant caveat in the limitations section of their study. Importantly, our finding that the finite N error was dominant in most of the literature studies does not invalidate the respective study results (nor those of other studies, for example in the field of quantitative imaging). It is rather a call for considering the finite N error and reporting the estimated std(AUC).

The scaling law $N_{\text{equality of errors}}\propto {CoV}^{-\text{4}}$ is intriguing. It predicts that $N_{\text{equality of errors}}$ increases quickly as the CoV decreases. This entails the need for caution when interpreting the $N_{\text{equality of errors}}$ found in our (or any) analysis. A small change in CoV can lead to substantially different $N_{\text{equality of errors}}$ values. This is important because the reported CoV values differ substantially among the available publications (see Table 3). For the smallest reported CoV of 3.2% [41], our analysis yields $N_{\text{equality of errors}}\approx$ 28,000, a number that is hardly ever reached in clinical MRI studies [5]. For the largest reported CoV of 20% [42], our analysis yields $N_{\text{equality of errors}}\approx$ 19, a sample size much smaller than is used in typical MRI studies [5]. Thus, an improve in CoV using better methodology will generally lead to much larger $N_{\text{equality of errors}}$ values (which may justify the use of larger sample sizes).

We only considered one use case, DWI of the breast involving ADC-based characterizations of the lesion type. However, considering that the size of errors is of similar magnitude in applications of DWI to other disease types [61,62], we suspect that our findings generalize to most other DWI studies and presumably also to many MRI studies in general, wherein finite N errors are likely to be dominant.

Our work has several limitations. For simplicity, we only assumed Gaussian distributions for the ADC values reported in the published studies. This limitation could be overcome if the circumstances demand it, potentially by using numerical approaches and retrieving the individual data points from a published study. Some studies could not be included because some key parameters such as standard deviations or coefficients of variation were not reported. This highlights a potential broader issue in the field, where insufficient reporting of variability may limit reproducibility and secondary analyses. Moreover, we focused on precision errors; inaccuracy errors could be treated similarly by calculating ${\Delta AUC}_{\text{inaccuracy}}$ from $p_{n,\text{inaccuracy}}(q)=p_{n}\left(q-q_{\text{inaccuracy}}\right)$. Our focus on precision errors is a limitation, as it ignores potential inaccuracy errors, e.g., systematic deviations between measured and true ADC values. While these were assumed negligible, future studies could explicitly model both error types, for example using phantom calibrations or multicenter datasets to assess inter-scanner bias. Another limitation of this study is that literature data were modeled assuming normal distributions. However, this reflects data availability and was chosen to preserve distributional features where individual data were accessible.

In conclusion, we found that finite N errors were generally dominant in the analyzed test case of DWI of the female breast. Based on our study, it appears worthwhile to consider the relative magnitudes of various error types when planning or evaluating studies involving quantitative parameters.

## Supporting information

S1 File Additional information. (DOCX)

S2 File Matlab code used to generate Figs 4–6, and supporting figures. (M)

S3 File Matlab code to perform your own analysis. (M)

S4 File Function for ROC analysis. (M)

S5 File ADC values of in-house study. (MAT)

S6 File Function AUC from PDFs. (M)

## Abbreviations

ADC: apparent diffusion coefficient
AUC: area under the receiver operating characteristic curve
BI-RADS: Breast Imaging Reporting and Data System
COV: coefficient of variation
DWI: diffusion-weighted imaging
MRI: magnetic resonance imaging
PDF: probability density function
Std: standard deviation
