# Supplementary material for: Finite sample size errors in the context of multiple error sources in quantitative medical imaging: An evaluation for breast magnetic resonance diffusion-weighted imaging
Source: PLoS One. 2026 Jun 4;21(6):e0341201. doi: 10.1371/journal.pone.0341201 (PMC13235925; doi:10.1371/journal.pone.0341201)
Supplement: S1 File — (DOCX) [file pone.0341201.s001.docx]

**Supporting Information**

**Detailed characteristics of the in-house-study**

**Inclusion/Exclusion criteria**

Exclusion criteria were histopathological findings available only for lymph nodes (n = 6), poor visibility on a DWI sequence (n = 6), nonvisible microcalcifications (n = 6), breast implants on the lesion’s side (n = 4), motion artifacts (n = 2), predominant cysts (n = 1), and insufficient histopathological findings (n = 1) (see S1 Fig).

Finally, 147 women with 171 breast lesions (83 benign, 88 malignant) were included in the study. A total of 123 women had exactly one either benign or malignant finding. 24 women had exactly two findings, including 8 women with either two benign or two malignant findings. 16 women had both a benign and a malignant finding (see S1 Table).


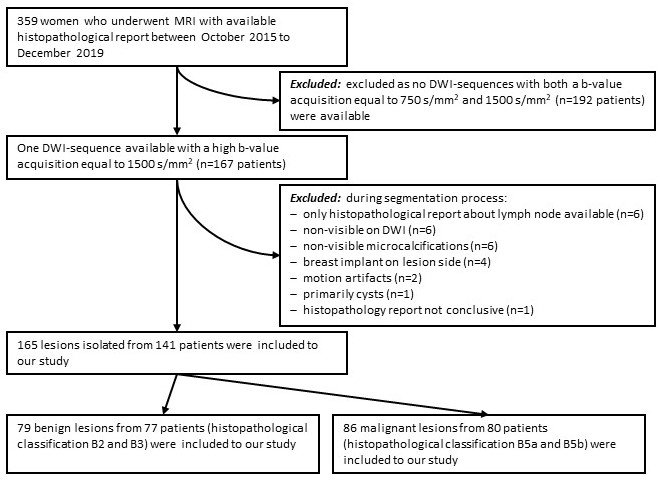


**S1 Fig. Flow chart showing inclusion and exclusion criteria for the in-house study.**

Among 359 women previously examined between 2015 and 2019, a total of 171 breast lesions from 147 patients were included based on image quality and histopathological confirmation.

DWI = diffusion-weighted imaging. ADC = apparent diffusion coefficient.

**S1 Table. Demographic characteristics of the in-house study population, stratified by lesion type (benign versus malignant).**

|  | Complete patient cohort (lesions) | Women with benign findings (lesions) | Women with malignant findings (lesions) | p value* |
| --- | --- | --- | --- | --- |
| Number of women with findings | 147 (171 lesions) | 81 (83 benign lesions) | 82 (88 malignant lesions) |  |
| Number of women with a single lesion | 123 (123 lesions) | 63 (63 benign lesions) | 60 (60 malignant lesions) |  |
| Number of women with two lesions | 24 (48 lesions) | 18 (20 benign lesions) | 22 (28 malignant lesions) |  |
| Number of women with two lesions of the same dignity | 8 (16 lesions) | 2 (4 benign lesions) | 6 (12 malignant lesions) |  |
| Number of women with both one benign and one malignant finding | 16 (32 lesions) | 16 (16 benign lesions) | 16 (16 malignant lesions) |  |
| Examination period | March 2017 – December 2019 | March 2017 – December 2019 | March 2017 – December 2019 |  |
| Age (y)  Mean ± std  Median  Min  Max | 53 ± 13  53  22  86 | 49 ± 12  50  22  81 | 57 ± 12  58  30  86 | < .001 |
| Patient size [m]  Mean ± std  Median  Min  Max | 1.66 ± 0.07  1.65  1.48  1.88 | 1.66 ± 0.07  1.66  1.49  1.88 | 1.65 ± 0.06  1.65  1.48  1.79 | .29 |
| Patient weight [kg]  Mean ± std  Median  Min  Max | 70.99 ± 14.7  68  43  150 | 69.34 ± 11.83  70  48  99 | 72.48 ± 16.34  67.5  43  150 | .4 |

std = standard deviation.

* Indicates p value by two-tailed Wilcoxon-Rank-Test for women with benign versus women with malignant findings.

**Histopathological Analysis**

To ensure sufficient quality of this study, only lesions in which histological diagnostic of the target lesion was available were included.

Of the 171 lesions, 152 were punches and 19 were vacuum biopsies. Histopathologic classification according the European guidelines for quality assurance in breast cancer screening and diagnosis (1) was performed. Classifications B2 and B3 were considered benign for this study, and classifications B5a and B5b were considered malignant.

S2 Table shows the assignment of radiologic classification according to ACR BI-RADS (2) to the side-specific histopathologic classification as benign or malignant. All 10 lesions classified as BI-RADS 3 were histopathologically classified as benign. Out of 58 BI-RADS 5 lesions, 14 % (8/58) were histopathologically benign, and 86 % (50/58) were malignant. The 11 lesions classified as BI-RADS 6 were histopathologically benign in 18 % (2/11) and malignant in 82 % (9/11) of cases. In this regard, it should be noted that the BIRADs classification is assigned for the entire chest. In the cases considered herein, conspicuous lesions were therefore punched in addition to the previously histopathologically confirmed carcinoma to determine whether it was a satellite focus. In 16 cases, either BI-RADS 0 was assigned (histopathologically 75 % benign (9/12) and 25 % malignant lesions (3/12)), biopsy of a BI-RADS 2 lesion was performed following clinical consensus (histopathologically 100 % (3/3) benign lesions), or radiologically described lesions with unclear BI-RADS classification but existing histopathologic findings (histopathologically 50 % (1/2) benign and 50 % (1/2) malignant lesion). To avoid bias, no repeated scoring was conducted in these cases of unclear BI-RADS. Both invasive ductal carcinomas and ductal carcinomas in situ were included. When multiple classification categories were present in a patient, the highest grading was used for the lesion. Despite the malignancy of the breast site already being known, histopathological findings of satellite foci of known breast carcinomas were also used.

**S2 Table. Histopathological classification of lesions by BI-RADS level in the in-house study.**

| BI-RADS-classification (of MRI report) | histopathologically benign lesions (n = 79) | histopathologically malignant lesions (n = 86) |
| --- | --- | --- |
| 3 | 10/10 (100) | 0/10 (0) |
| 4 | 50/75 (67) | 25/75 (33) |
| 5 | 8/58 (14) | 50/58 (86) |
| 6 | 2/11 (18) | 9/11 (82) |
| not available or others* | 13/17 (76) | 4/17 (24) |

BI-RADS = Breast Imaging Reporting and Data System. Unless otherwise specified, data are numerators and denominators, with percentages in parentheses.

* Group includes lesions with BI-RADS 0 and radiologically BI-RADS 2 by clinical consensus.

**Imaging Protocol**

S3 Table provides details concerning different settings on which the DWI sequences of this study were based. All breast MRI examinations were performed with clinical indication on the hospital's routine MRI scanners. Standardized DWI MRIs of the 147 patients were performed on Siemens Skyra_fit scanners at 3 T (n = 117) or Siemens Aera at 1.5 T (n = 30) from Siemens Healthineers, Erlangen, Germany, with fat suppression. Routine MRI protocols consisted of morphological, T2-weighted, dynamic contrast-enhanced T1-weighted, and DWI sequences. DWI and ADC map acquisitions were performed with different sequence types and b-values, with b = 0/1500 s/mm^2^ (n = 6) and b = 0/750/1500 s/mm^2^ (n = 141) being common. Contrast agent-enhanced data were available for 122 of 141 patients. Gadovist was used 121 times, and Dotarem was used once. For all 147 patients, the mean echo time was 65 ms (range: 61-66 ms); the mean inversion time was 222 ms (range: 170-250 ms); the mean repetition time was 7,722 ms (range: 6,290-11,250 ms); the number of averages was 3; the acquisition matrix was 128 x 80, and the field of view range was (218-251 mm) x (350-400 mm). The slice thickness ranged from 3.5 to 4 mm.

All DWI images were pseudoanonymized by L.A.K. and transferred to a research workstation. Over a three-month period, the images were evaluated by a medical research student, J.V.E., with two years of experience in breast lesion segmentation. The segmentations were performed without knowledge of the histopathologic results while being aware of the BI-RADS classification and radiologic findings. All image analysis was performed under the supervision of a qualified radiologist with over 10 years of experience, S.B.

In alignment with the radiological findings, lesions were identified using a T1-weighted, post-contrast subtraction sequence. The entire volume of visible lesions, including soft foci, were manually segmented on the diffusion sequence with b = 1500 s/mm^2^. The border of the lesion was used as the region of interest, avoiding the inclusion of voxels that contained fat tissue. Slicer3D software (version 4.11.20210226) was used for this purpose. In addition, it was documented for which particular lesions histopathologic examination had been recommended and therefore represented the target lesion. Inconclusive lesions such as mastopathy areas and non-mass-enhancements were segmented with restraint to avoid distortion of the measurement (see Fig 1).

Subsequently, the exact matching of individual lesions to the histopathological findings was undertaken. In case of uncertainties, the lesion from which the tumor conglomerate had most likely evolved was selected. Strict attention was paid to separation between BI-RADS and histopathologic B-classification. A class-balance was aimed for to sufficiently establish future reference values.

3D Slicer software (version 4.11.20210226) was also used to calculate lesion size, voxel size, and quantitative ADC values. Measurements were obtained on ADC maps created from DWI sequences with b = 1500 s/mm^2^ and within the entire, three-dimensional lesion segmentation (see S4 Table). A t1-weighted, post-contrast subtraction sequence was used as the scalar volume.

To ensure that measurements were taken exactly in the lesion avoiding fat tissue, it was necessary to visually detect some shifts that had occurred during dynamic co-registration between the DWI sequence with b = 1500 s/mm^2^ and the ADC map, and to correct the segmentation manually.

**S3 Table. Settings of diffusion-weighted sequences.**

|  | s |
| --- | --- |
| b -values [s/mm^2^] | 50/750/1500 (n = 141)  50/1500 (n = 6) |
| TE [ms] *# | 65 (61-66) |
| TI [ms] *# | 222 (170-250) |
| Magnetic field strength [T] | Range: 1.5 (n = 30) to 3 (n = 117) |
| Model | Siemens |
| Model name | Skyra_fit (n=117),  Aera (n=30) |
| NoA | 3 |
| TR [ms]*# | 7722 (6290-11250) |
| Acquisition matrix | 128 x 80 |
| FoV [mm] # | (218-251) x (350-400) |
| Fat suppression | IR/fat saturation |
| Slice thickness [mm] # | 3.5-4 |

TE = echo time, TI = inversion time, NoA = number of averages, TR = repetition time, FoV = field of view, IR = inversion recovery.

* Mean reported.

# Unless not otherwise specified, data in parenthesis are ranges.

**S4 Table. Characteristics of the segmentations of diffusion-weighted images and evaluation of the apparent diffusion coefficients.**

|  | Benign lesions (n = 83) | Malignant lesions (n = 88) |
| --- | --- | --- |
| ADC-values [µm²/ms]  Mean ± std  Median  Min  Max | 1.34 ± 0.31  1.34  0.67  2.21 | 0.79 ± 0.2  0.8  0.26  1.25 |
| Volume [cm^3^]  Mean ± std  Median  Min  Max | 0.27 ± 0.52  0.08  0.02  4.1 | 2.21 ± 4.81  0.5  0.03  34.63 |
| Number of voxels  Mean ± std  Median  Min  Max | 32.02 ± 63.04  10  2  499 | 263.25 ± 583.95  56  4  4211 |

ADC = apparent diffusion coefficient, std = standard deviation.

**Statistical Analysis**

All statistical analyses were performed by F.B.L., a physicist with over 10 years of experience in medical statistics, and by J.V.E. All codes and plots were generated using the software Matlab (version R2020b).

A characterization of our own data was performed with the software SigmaPlot (version 15.0) to assess whether it was a representative patient collective. To estimate the distribution between women with malignant or benign findings, the Shapiro-Wilk test was applied to age (participants with benign findings: p = .12; with malignant findings: p = .78), size (participants with benign findings: p = .06; with malignant findings: p = .42) and weight (participants with benign findings: p = .16; with malignant findings: p = .02. A two-tailed Wilcoxon rank test was conducted to assess significance. This yielded p < .001 for patient age, p = .29 for patient size, and p = .4 for patient weight. Significance was assumed at a p value less than or equal to .05. Therefore, for all three groups, a normal distribution approximation was assumed as well as a significant difference.

The voxel numbers of segmentations, their volumes, and the results of ADC acquisitions are listed in S4 Table. The mean ADC values were 1.34 ± 0.31 µm²/ms (range: 0.67-2.21 µm²/ms, median ADC: 1.34 µm²/ms) for the 83 benign lesions and 0.79 ± 0.2 µm²/ms (range: 0.26-1.25 µm²/ms, median ADC: 0.8 µm²/ms) for the 88 malignant lesions. The mean volume was 0.27 ± 0.52 cm³ (range: 0.02-4.1 cm³, median volume: 0.08 cm^3^) for the 83 benign lesions and 2.21 ± 4.81 cm³ (range: 0.03-34.63 cm³, median volume: 0.5 cm³) for the 88 malignant lesions. The mean voxel size was 32.02 ± 63.04 (range: 2-499, median number of voxels: 10) for the 83 benign lesions and 263.25 ± 583.95 (range: 4-4211, median number of voxels: 56) for the 88 malignant lesions.

The AUC for the entire data set was 0.92. The Youden index value was 0.69. The ADC-threshold was scored as 1.056 µm²/ms based on the Youden Index with 0.69 (sensitivity = 89 %; specificity = 81 %).

Limitations of the in-house-study

The choice and application of alternative b-values as well as variation in ADC map generation would produce different results than those derived from our in-house study. The ADC values for the in-house dataset were extracted as provided in the ADC map of the MRI scanner software, which included all b-values. This implies that in our case a b-value of 1500 s/mm^2^ was included in the calculation of the ADC values, introducing bias by including kurtosis effects. However, as in clinical routines the scanner-provided ADC maps are commonly used to assess ADC values of lesions, we maintained this “clinical” workflow. Furthermore, in the case of multiple lesions, it may not be possible to clearly differentiate which one was punched. Similarly, potential errors may result from challenging identifications of the correct target lesion. For example, if the main lesion was located prepectorally, the more ventrally located lesion may still have been punched to avoid the risk of pneumothorax. MRI-guided biopsy and clipping for better locating would potentially help to avoid these kinds of limitations. Thus, as in human error potential, in single cases segmentations could have unintentionally been performed on the wrong lesion instead of the previously reported target lesion.

Our in-house study included women who had both one benign and one malignant lesion. Therefore, since the malignant and benign groups were not independent, significance testing using generalized linear mixed models would have been required. However, because the in-house study was intended to serve simply as an applied example, we did not prioritize this test correction. For the same reason, we decided to simply assume a Gaussian distribution for our data. In comparison to previous studies, our low-stratified and not pre-selected patient collective, as well as including predefined b3-classification to our evaluation of benign ADCs, may have led to a comparably lower overall AUC of our in-house study. A definite approximation to a specific threshold value could not yet be derived for our study size with n = 171. Extended research with larger sample sizes is required to further improve the reliability and validity of our results, but these ambitions were beyond the scope of the current study.

The ideal case to evaluate the $CoV$ of our own study would have been a prospective assessment. In our retrospective study, the determination of a $CoV$ for was not feasible, as there were neither two different examination time points nor a repositioning accounting for the data selection.

**Literature search**

We used the literature database PubMed and the search term "Mamma AND DWI AND ADC" in December 2021. The retrospective search yielded n = 174 studies published between 2002 and 2021. The studies were then selected according to the following exclusion criteria (see Fig 2). To make the findings comparable to our in-house study (c.f. below), we excluded studies that used MRI scanners from a different vendor (n = 87) or that used older scanners from the same vendor (i.e., market introduction before 2001, n = 11). The comparability of the studies was ensured regarding the technical developments; this was why primarily last-generation devices were included. We wanted to focus on the task of differentiating benign and malignant breast lesions and thus excluded studies that sought to differentiate with respect to receptor status, histopathological grade, cancer type, tumor size, or pre- or post-contrast agent (n = 17). In addition, studies were excluded if ADC values were not available for both benign and malignant lesions (n = 15) or if fewer than 50 lesions had been included (n = 5). Studies were also excluded if we rated the ADC calculation as missing, insufficient, or nontransparent (n = 13) or if the stated DWI sequence parameters were not sufficiently defined (n = 2). Eventually, we included 24 studies (3-26). Table 1 summarizes the mean and standard deviations of ADC values of benign and malignant lesions reported in those studies including the following adaptions after a sanity check of the reported values:

- Study 3 by Ohlmeyer et al. (5): The study only reported the standard deviations of the ADC values in the considered region of interest. A reevaluation of this study, which is from our host institute, was performed. The standard deviations of the ADC values were computed for each of the two classes (benign and malignant class) and used in the current study.
- Study 7 by Fan et al. (9): The reported standard deviations were much smaller than in the other studies (0.019 µm²/ms and 0.110 µm²/ms). These small standard deviations were also not in keeping with the reported $AUC$ values. We assumed that they presumably had reported the standard error of the mean ADC and thus rescaled these standard deviations with $\sqrt{N_{1}}$ and $\sqrt{N_{2}}$, where $N_{1}$ and $N_{2}$ are the sample sizes of the two groups.
- Study 10 by Yamaguchi et al. (12): They had performed an evaluation with two sequences (single-shot EPI and readout-segmented EPI). We used the single-shot EPI data, which is closer to what the other studies did. They reported the following standard deviations. Single-shot EPI: benign 2.06 µm²/ms, malignant 0.22 µm²/ms. Readout-segmented: benign: 0.22 µm²/ms, malignant 0.22 µm²/ms. We assumed that the reported value 2.06 µm²/ms was a typo and used 0.21 µm²/ms instead.
- Study 14 by Akin et al. (16): They reported the following standard deviations for benign and malignant class: 2.388 µm²/ms and 0.171 µm²/ms. We again assumed that the large value 2.388 µm²/ms was a typo (as it also did not match to the reported $AUC$ values) and changed it into 0.24 µm²/ms.

To collect data about test/retest coefficients of variation for breast DWI, the literature database PubMed was used with the search term “coefficient of variation AND “breast OR mamma” AND ADC AND DWI” in December 2021. Studies were included if they listed DWI- or DTI-derived breast ADC examinations and assessments of test/retest $CoV$. Ten studies published between 2001 and 2021 with sample sizes ranging from 8 to 76 were included. Further inclusion criteria were a precise description of the dignity of the tissue examined and publication of the numerical value of the $CoV$ obtained. If indicated, the method of calculation of the $CoV$, both the chronological sequence of measurements and the use of the formula itself, was included. Studies with 2D and 3D segmentations were included. If not reported, we $CoVs$ from the reported ADCs and their standard deviations. For those studies that did not report a mean $CoV$ but only a range, the mean was set to the mean of the reported range limits. Table 4 summarizes test/retest $CoVs$ reported for breast DWI.

**Area under the curve (AUC) derived from Gaussian PDFs**

Here were summarize some formulas to support the Methods section of the main text. Given the probability density functions (PDFs) $p_{1}\left( q \right)$ and $p_{2}\left( q \right)$ corresponding to positive and negative with $q_{\mathrm{thresh}}$ as cutoff value between the two classes, respectively, and the cumulative distribution functions $P_{1}\left( q \right)$ and $P_{2}\left( q \right)$, the true positive rate $\mathrm{TPR}$, the false negative rate $\mathrm{FNR}$, the false positive rate $\mathrm{FPR}$, and the true negative rate $\mathrm{TNR}$ are given by

$$\mathrm{TPR}= \int_{-\infty}^{q_{\mathrm{thresh}}} p_{1}\left( q \right)dq=P_{1}\left( q_{\mathrm{thresh}} \right),$$

$$\mathrm{FNR}= \int_{q_{\mathrm{thresh}}}^{\infty} p_{1}\left( q \right)dq=1-P_{1}\left( q_{\mathrm{thresh}} \right),$$

$$\mathrm{FPR}= \int_{-\infty}^{q_{\mathrm{thresh}}} p_{2}\left( q \right)dq=P_{2}\left( q_{\mathrm{thresh}} \right),$$

$$\mathrm{TNR}= \int_{q_{\mathrm{thresh}}}^{\infty} p_{2}\left( q \right)dq=1-P_{2}\left( q_{\mathrm{thresh}} \right).$$

The sensitivity, specificity, and Youden index $J$ are given by

$$\mathrm{sensitivity}=\frac{\mathrm{TPR}}{TPR+FNR}=\frac{P_{1}\left( q_{\mathrm{thresh}} \right)}{P_{1}\left( q_{\mathrm{thresh}} \right)+1-P_{1}\left( q_{\mathrm{thresh}} \right)}=P_{1}\left( q_{\mathrm{thresh}} \right),$$

$$\mathrm{specificity}=\frac{\mathrm{TNR}}{TNR+FPR}=\frac{1-P_{2}\left( q_{\mathrm{thresh}} \right)}{1-P_{2}\left( q_{\mathrm{thresh}} \right)+P_{2}\left( q_{\mathrm{thresh}} \right)}=1-P_{2}\left( q_{\mathrm{thresh}} \right),$$

For normally distributed data centered at 0 and 1 with standard deviations $\sigma_{1}$ and $\sigma_{2}$, the PDFs and cumulative distribution functions are

$$p_{1}\left( q \right)=\frac{1}{\sqrt{2\pi}\sigma_{1}}\cdot\exp\left( -\frac{q^{2}}{2\sigma_{1}^{2}} \right),$$

$$p_{2}\left( q \right)=\frac{1}{\sqrt{2\pi}\sigma_{2}}\cdot\exp\left( -\frac{\left( q-1 \right)^{2}}{2\sigma_{2}^{2}} \right),$$

$$P_{1}\left( q \right)=\frac{1}{2}\cdot\left( 1+\mathrm{erf} \left( \frac{q}{\sqrt{2}\sigma_{1}} \right) \right),$$

$$P_{2}\left( q \right)=\frac{1}{2}\cdot\left( 1+\mathrm{erf} \left( \frac{-1+q}{\sqrt{2}\sigma_{2}} \right) \right),$$

where $\mathrm{erf}$ is the error function.

The area under the curve $AUC$ is given by

$$AUC=\int_{0}^{1} \mathrm{sensitivity}\left( q \right)*d\left( 1-specificity\left( q \right) \right)=\int_{0}^{1} P_{1}\left( q \right)dP_{2}\left( q \right)$$

$$=\int_{-\infty}^{\infty} P_{1}\left( q \right)\frac{dP_{2}\left( q \right)}{dq}dq=\int_{-\infty}^{\infty} P_{1}\left( q \right)p_{2}\left( q \right)dq$$

$$=\int_{-\infty}^{\infty} \frac{1}{2}\cdot\left( 1+\mathrm{erf} \left( \frac{q}{\sqrt{2}\sigma_{1}} \right) \right)\frac{1}{\sqrt{2\pi}\sigma_{2}}\cdot\exp\left( -\frac{\left( q-1 \right)^{2}}{2\sigma_{2}^{2}} \right)dq$$

$$=\int_{-\infty}^{\infty} \frac{1}{2}\cdot\frac{1}{\sqrt{2\pi}\sigma_{2}}\cdot\exp\left( -\frac{\left( q-1 \right)^{2}}{2\sigma_{2}^{2}} \right)dq+\int_{-\infty}^{\infty} \frac{1}{2}\cdot\mathrm{erf} \left( \frac{q}{\sqrt{2}\sigma_{1}} \right)\frac{1}{\sqrt{2\pi}\sigma_{2}}\cdot\exp\left( -\frac{\left( q-1 \right)^{2}}{2\sigma_{2}^{2}} \right)dq$$

$$=\frac{1}{2}+\frac{1}{2}\int_{-\infty}^{\infty} \mathrm{erf} \left( \frac{q}{\sqrt{2}\sigma_{1}} \right)\frac{1}{\sqrt{2\pi}\sigma_{2}}\cdot\exp\left( -\frac{\left( q-1 \right)^{2}}{2\sigma_{2}^{2}} \right)dq$$

A solution of this integral exists (27):

$$\int_{-\infty}^{\infty} \mathrm{erf} \left( aq+b \right)\frac{1}{\sqrt{2\pi}\sigma^{2}}\cdot\exp\left( -\frac{\left( q-\mu\right)^{2}}{2\sigma^{2}} \right)dq=\mathrm{erf} \left( \frac{a\mu+b}{\sqrt{1+2a^{2}\sigma^{2}}} \right)$$

By setting $a=\frac{1}{\sqrt{2}\sigma_{1}}$, $b=0$, $\mu=1$, $\sigma=\sigma_{2}$, we find

$$AUC=\frac{1}{2}+\frac{1}{2}\mathrm{erf} \left( \frac{\frac{1}{\sqrt{2}\sigma_{1}}}{\sqrt{1+2\frac{\sigma_{2}^{2}}{2\sigma_{1}^{2}}}} \right)=\frac{1}{2}+\frac{1}{2}\mathrm{erf} \left( \frac{1}{\sqrt{2\sigma_{1}^{2}}\sqrt{1+\frac{\sigma_{2}^{2}}{\sigma_{1}^{2}}}} \right)=\frac{1}{2}+\frac{1}{2}\mathrm{erf} \left( \frac{1}{\sqrt{2\sigma_{1}^{1}+2\sigma_{2}^{2}}} \right)$$

**Imprecision**

As described in the main text, we assume a Gaussian error with standard deviation $\sigma_{q}$, which is described by

$$p_{\mathrm{imprecision}}\left( q \right)=\frac{1}{\sqrt{2\pi}\sigma_{q,CoV}}\cdot\exp\left( -\frac{q^{2}}{2\sigma_{q,CoV}^{2}} \right)$$

The respective PDFs of the two classes are (where $*$ denotes the convolution operation):

$$p_{1,imprecision}\left( q \right)=p_{1}\left( q \right)*p_{\mathrm{imprecision}}\left( q \right) =\frac{1}{\sqrt{2\pi}\cdot\sqrt{\sigma_{1}^{2}+\sigma_{q,CoV}^{2}}}\cdot\exp\left( -\frac{q^{2}}{2\cdot\left( \sigma_{1}^{2}+\sigma_{q,CoV}^{2} \right)} \right)\mathcal{=N}\left( q,0,\sigma_{1}^{2}+\sigma_{q,CoV}^{2} \right)$$

$$p_{2,imprecision}\left( q \right)\mathcal{=N}\left( q,0,\sigma_{2}^{2}+\sigma_{q,CoV}^{2} \right).$$

**Visualization of the normalization**

S2 Fig visualizes the effect of the normalization procedure on the PDFs (Eqs. 1-4). The shape of the PDFs and their overlap do not change, but their means are shifted to 0 and 1, respectively, by the normalization.


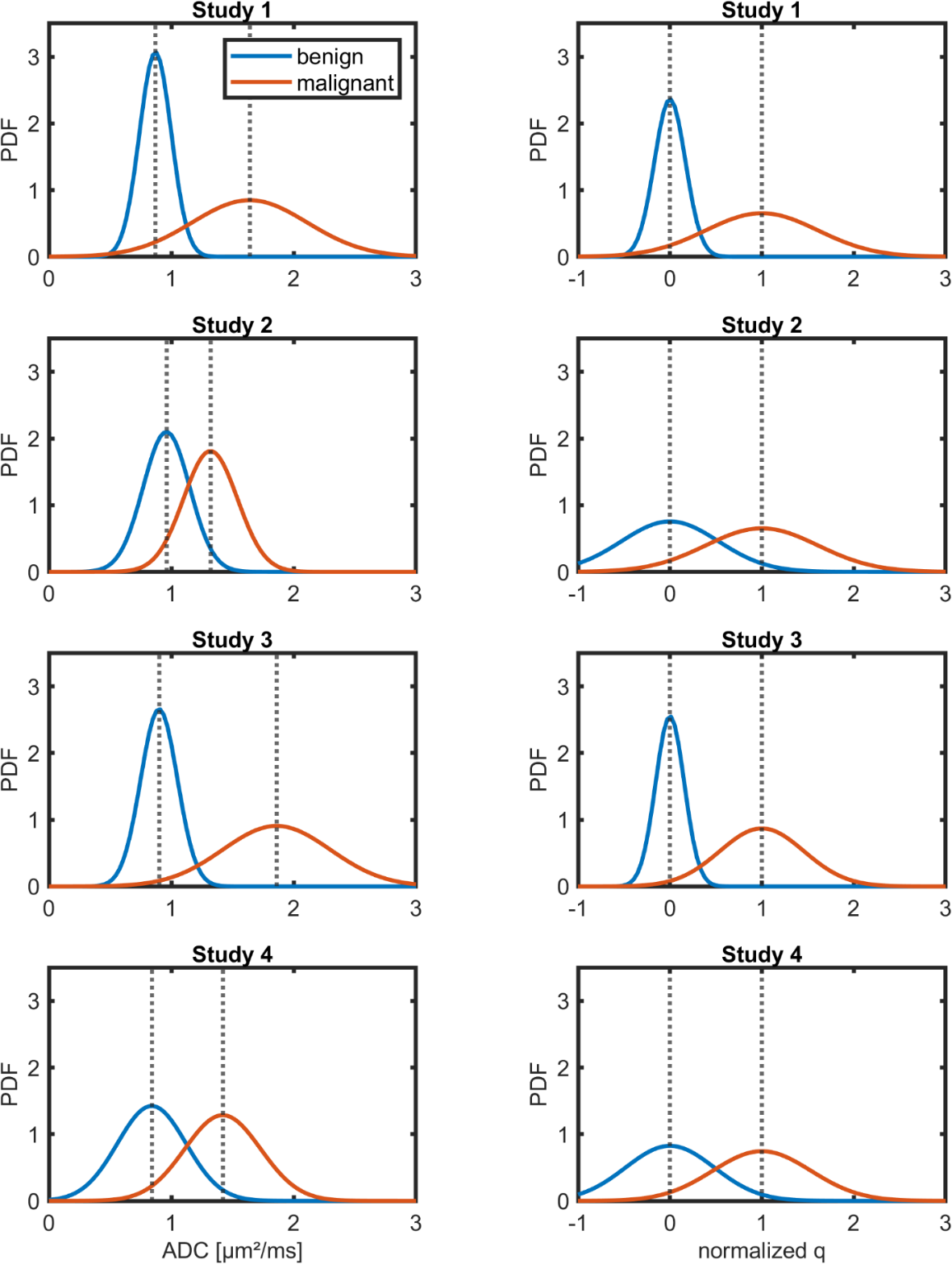


**S2 Fig. Illustration of the normalization procedure for the first four studies.** Vertical lines indicate the means, which are shifted to 0 and 1, respectively, by the normalization procedure.

**Influence of the b-value**

To estimate the effect of the used b-values, we retrieved the b-values from the considered studies (see S5 Table).

In some studies, it was not clear to us, which b-values were actually used for the computation of the ADC. In study 1 (3), we did not find a statement on whether the b-value 1500 s/mm² was used for the ADC calculation. In study 2 (4), we were uncertain, whether further b-values had been acquired. In study 7 (9), we did not find a statement on whether the b-value 0 s/mm² was used for the ADC calculation (there is an option in this scanner’s software version that allows including or excluding small b-values). We thus do not consider these studies further here.

Some studies used a middle b-value of 400 s/mm² or 500 s/mm². Since this middle b-value has little effect on the ADC (28), we ignored the presence of these middle b-values.

Some studies used b-values 50-250 s/mm² and 800-1000 s/mm². In our analysis, we grouped them together (Group 1). Some studies used the b-values 0 s/mm² and 750-1000. For these studies, the intravoxel incoherent motion (IVIM) effect was thus relevant (29). We grouped these studies together (Group 2). Additionally, two studies used a b-values of 1500 s/mm² for the ADC calculation (Group 3).

To isolate the effect of the b-values, and to minimize the effect of the varying sample sizes, we multiplied $\mathrm{std}\left( AUC \right)$ with $\sqrt{N/100}$ in order to effectively set the sample size to a virtual value of 100.

S3 Fig shows a scatter plot of the AUC values, the $\mathrm{std}\left( AUC \right)\cdot\sqrt{N/100}$ values, and the ${\Delta AUC}_{\mathrm{precision}}$ values. The differences between group 1 and 2 appear to be small. Group 3 stands out with lower error values, but this group is small and contains only two studies.

**S5 Table. Influence of b-values**

| **Study** | $\boldsymbol{N}$ | $\mathbf{std}\left( \boldsymbol{AUC} \right)$  (%) | $\mathbf{std}\left( \boldsymbol{AUC} \right)\boldsymbol{\cdot}\sqrt{\boldsymbol{N/100}}$  (%) | $\boldsymbol{AUC}$ | ${\boldsymbol{\Delta}\boldsymbol{AUC}}_{\mathbf{precision}}$  (%) | **b-values**  (s/mm²) | **Group** |
| --- | --- | --- | --- | --- | --- | --- | --- |
| 1 (3) | 131 | 2.44 | 2.79 | 0.943 | 0.70 | 50, 800, 1500* |  |
| 2 (4) | 246 | 2.08 | 3.26 | 0.892 | 1.96 | 0, 1000, potentially further b-values* |  |
| 3 (5) | 72 | 2.06 | 1.75 | 0.981 | 0.53 | 50, 800 | 1 |
| 4 (6) | 213 | 2.03 | 2.96 | 0.917 | 0.90 | 50, 400 or 500, 1000 | 1 |
| 5 (7) | 116 | 5.45 | 5.87 | 0.645 | 0.76 | 50, 800 | 1 |
| 6 (8) | 210 | 2.63 | 3.81 | 0.885 | 0.93 | 50, 850 | 1 |
| 7 (9) | 89 | 6.47 | 6.10 | 0.853 | 0.65 | 0, 50, 800* |  |
| 8 (10) | 144 | 4.50 | 5.40 | 0.808 | 1.50 | 0, 750 | 2 |
| 9 (11) | 95 | 3.70 | 3.61 | 0.867 | 2.28 | 50, 800 | 1 |
| 10 (12) | 80 | 0.52 | 0.46 | 0.995 | 0.74 | 0, 1000 | 2 |
| 11 (13) | 61 | 1.08 | 0.84 | 0.988 | 0.95 | 0, 200, 700, we used ADC_0,700_ | 2 |
| 12 (14) | 56 | 4.14 | 3.10 | 0.903 | 1.01 | 0, 1000 | 2 |
| 13 (15) | 85 | 1.41 | 1.35 | 0.976 | 1.16 | 50, 400, 800 | 1 |
| 14 (16) | 181 | 1.08 | 1.45 | 0.971 | 1.01 | 50, 500, 800 | 1 |
| 15 (17) | 104 | 4.22 | 4.30 | 0.843 | 1.06 | 50, 400, 800 | 1 |
| 16 (18) | 326 | 1.42 | 2.56 | 0.962 | 1.51 | 0, 500, 1000 | 2 |
| 17 (19) | 170 | 2.49 | 3.25 | 0.892 | 0.88 | 50, 400, 800 | 1 |
| 18 (20) | 98 | 3.67 | 3.63 | 0.889 | 1.44 | 50, 1000 | 1 |
| 19 (21) | 72 | 1.30 | 1.10 | 0.981 | 1.24 | 0, 700 | 2 |
| 20 (22) | 169 | 4.08 | 5.30 | 0.758 | 0.64 | 0, 100, 500, 800, 1000 | 2 |
| 21(23) | 115 | 6.16 | 6.61 | 0.683 | 0.49 | 50, 800, 1500, ADC computed with 50 and 1500 | 3 |
| 22(24) | 106 | 2.07 | 2.13 | 0.970 | 1.55 | 0, 250, 500, 750, 1000, ADC computed with b$\geq$250 | 1 |
| 23 (25) | 61 | 3.90 | 2.50 | 0.933 | 1.39 | 0, 50, 100, 250, 400, 550, 700, 850, 1000, 1250, we used the ADC computed with all b-values | 2 |
| 24 (26) | 111 | 4.15 | 4.37 | 0.829 | 0.55 | 500, 1000, 1500, 2000, 3000, ADC computed with 500 and 1500 | 3 |

AUC = area under the curve; $\mathrm{std}\left( AUC \right)$ = finite N error; ${\Delta AUC}_{\mathrm{precision}}$ = precision error; MC = Monte-Carlo Simulation; PDF = probability density function; $std$ = standard deviation. * = not stated which b-values were used for the ADC computation.


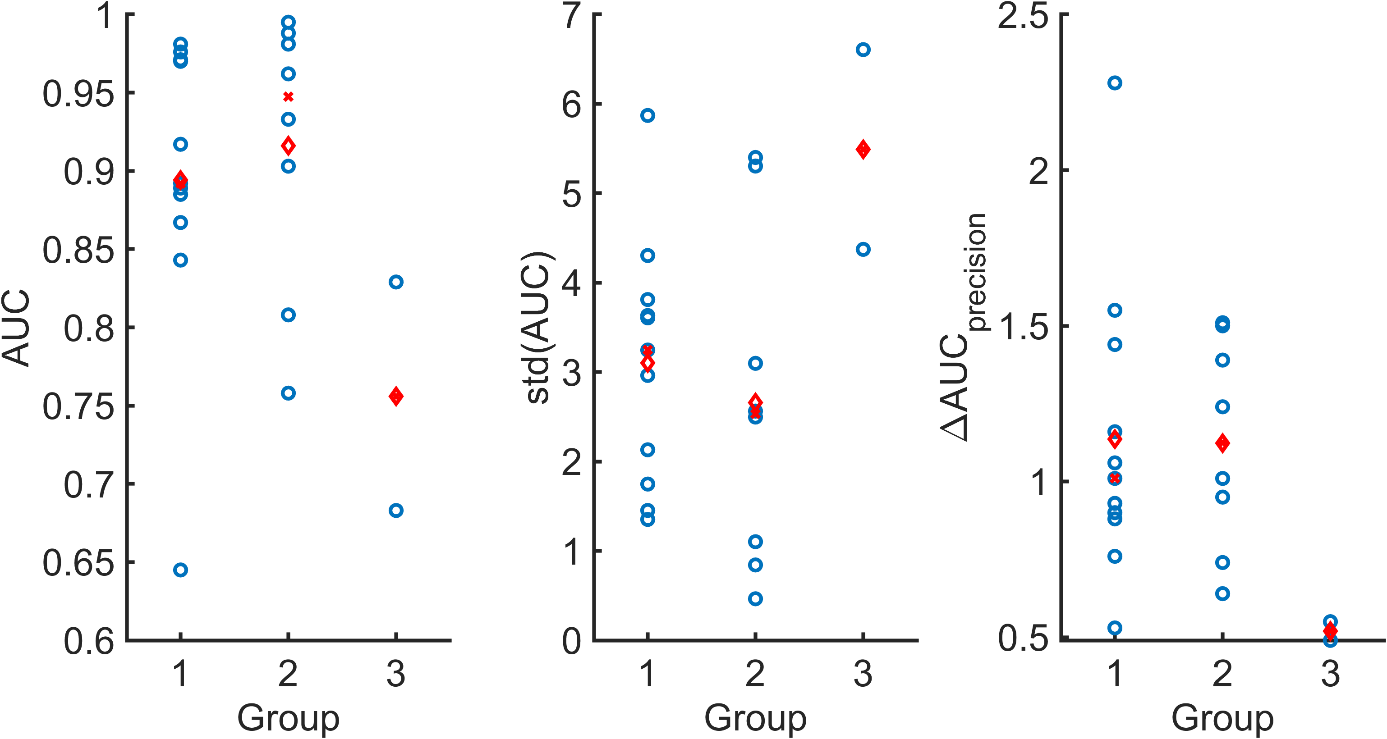


**S3 Fig. Illustration of the b-value analysis.** Blue circles represent the individual studies, the red diamonds the means and the red crosses the medians. $\mathrm{std}\left( AUC \right)$ is plotted for the virtual sample size of 100.

**References**

1. Perry N, Broeders M, De Wolf C, Törnberg S, Holland R, Von Karsa L. European guidelines for quality assurance in breast cancer screening and diagnosis. Fourth edition—summary document. Annals of Oncology. 2008;19(4):614-22.

2. Morris E, Comstock C, Lee C. ACR BI-RADS® Magnetic Resonance Imaging. ACR BI-RADS® Atlas, Breast Imaging Reporting and Data System. Reston, VA: American College of Radiology; 2013.

3. Yadav P, Harit S, Kumar D. Efficacy of high-resolution, 3-D diffusion-weighted imaging in the detection of breast cancer compared to dynamic contrast-enhanced magnetic resonance imaging. Polish Journal of Radiology. 2021;86(1):277-86.

4. Duran B, Agridag Ucpinar B. Four Different Apparent Diffusion Coefficient Measurement Methods in Breast Masses. J Coll Physicians Surg Pak. 2021;31(9):1024-9.

5. Ohlmeyer S, Laun FB, Palm T, Janka R, Weiland E, Uder M, et al. Simultaneous Multislice Echo Planar Imaging for Accelerated Diffusion-Weighted Imaging of Malignant and Benign Breast Lesions. Invest Radiol. 2019;54(8):524-30.

6. Kul S, Metin Y, Kul M, Metin N, Eyuboglu I, Ozdemir O. Assessment of breast mass morphology with diffusion-weighted MRI: Beyond apparent diffusion coefficient. Journal of Magnetic Resonance Imaging. 2018;48(6):1668-77.

7. Chen Y, Wu B, Liu H, Wang D, Gu Y. Feasibility study of dual parametric 2D histogram analysis of breast lesions with dynamic contrast-enhanced and diffusion-weighted MRI. Journal of Translational Medicine. 2018;16(1).

8. Zhang M, Horvat JV, Bernard-Davila B, Marino MA, Leithner D, Ochoa-Albiztegui RE, et al. Multiparametric MRI model with dynamic contrast-enhanced and diffusion-weighted imaging enables breast cancer diagnosis with high accuracy. Journal of Magnetic Resonance Imaging. 2019;49(3):864-74.

9. Fan WX, Chen XF, Cheng FY, Cheng YB, Xu T, Zhu WB, et al. Retrospective analysis of the utility of multiparametric MRI for differentiating between benign and malignant breast lesions in women in China. Medicine (Baltimore). 2018;97(4).

10. An YY, Kim SH, Kang BJ. Differentiation of malignant and benign breast lesions: Added value of the qualitative analysis of breast lesions on diffusion-weighted imaging (DWI) using readout-segmented echo-planar imaging at 3.0 T. PLOS ONE. 2017;12(3):e0174681.

11. Liu H-L, Zong M, Wei H, Lou J-J, Wang S-Q, Zou Q-G, et al. Preoperative predicting malignancy in breast mass-like lesions: value of adding histogram analysis of apparent diffusion coefficient maps to dynamic contrast-enhanced magnetic resonance imaging for improving confidence level. The British Journal of Radiology. 2017;90(1079):20170394.

12. Yamaguchi K, Nakazono T, Egashira R, Komori Y, Nakamura J, Noguchi T, et al. Diagnostic Performance of Diffusion Tensor Imaging with Readout-segmented Echo-planar Imaging for Invasive Breast Cancer: Correlation of ADC and FA with Pathological Prognostic Markers. Magnetic Resonance in Medical Sciences. 2017;16(3):245-52.

13. Teruel JR, Goa PE, Sjøbakk TE, Østlie A, Fjøsne HE, Bathen TF. A Simplified Approach to Measure the Effect of the Microvasculature in Diffusion-weighted MR Imaging Applied to Breast Tumors: Preliminary Results. Radiology. 2016;281(2):373-81.

14. Jiang R, Zeng X, Sun S, Ma Z, Wang X. Assessing Detection, Discrimination, and Risk of Breast Cancer According to Anisotropy Parameters of Diffusion Tensor Imaging. Medical Science Monitor. 2016;22:1318-28.

15. Onaygil C, Kaya H, Ugurlu MU, Aribal E. Diagnostic performance of diffusion tensor imaging parameters in breast cancer and correlation with the prognostic factors. Journal of Magnetic Resonance Imaging. 2017;45(3):660-72.

16. Akin Y, Ugurlu MU, Kaya H, Aribal E. Diagnostic Value of Diffusion-weighted Imaging and Apparent Diffusion Coefficient Values in the Differentiation of Breast Lesions, Histpathologic Subgroups and Correlatıon with Prognostıc Factors using 3.0 Tesla MR. Journal of Breast Health. 2016;12(3):123-32.

17. Spick C, Pinker-Domenig K, Rudas M, Helbich TH, Baltzer PA. MRI-only lesions: application of diffusion-weighted imaging obviates unnecessary MR-guided breast biopsies. European Radiology. 2014;24(6):1204-10.

18. Sharma U, Sah RG, Agarwal K, Parshad R, Seenu V, Mathur SR, et al. Potential of Diffusion-Weighted Imaging in the Characterization of Malignant, Benign, and Healthy Breast Tissues and Molecular Subtypes of Breast Cancer. Frontiers in Oncology. 2016;6.

19. Ertas G, Onaygil C, Akin Y, Kaya H, Aribal E. Quantitative differentiation of breast lesions at 3T diffusion-weighted imaging (DWI) using the ratio of distributed diffusion coefficient (DDC). Journal of Magnetic Resonance Imaging. 2016;44(6):1633-41.

20. Sun K, Chen X, Chai W, Fei X, Fu C, Yan X, et al. Breast Cancer: Diffusion Kurtosis MR Imaging—Diagnostic Accuracy and Correlation with Clinical-Pathologic Factors. Radiology. 2015;277(1):46-55.

21. Teruel JR, Goa PE, Sjøbakk TE, Østlie A, Fjøsne HE, Bathen TF. Diffusion weighted imaging for the differentiation of breast tumors: From apparent diffusion coefficient to high order diffusion tensor imaging. Journal of Magnetic Resonance Imaging. 2016;43(5):1111-21.

22. Yoo H, Shin HJ, Baek S, Cha JH, Kim H, Chae EY, et al. Diagnostic performance of apparent diffusion coefficient and quantitative kinetic parameters for predicting additional malignancy in patients with newly diagnosed breast cancer. Magnetic Resonance Imaging. 2014;32(7):867-74.

23. Satake H, Nishio A, Ikeda M, Ishigaki S, Shimamoto K, Hirano M, et al. Predictive Value for Malignancy of Suspicious Breast Masses of BI-RADS Categories 4 and 5 Using Ultrasound Elastography and MR Diffusion-Weighted Imaging. American Journal of Roentgenology. 2011;196(1):202-9.

24. Inoue K, Kozawa E, Mizukoshi W, Tanaka J, Saeki T, Sakurai T, et al. Usefulness of diffusion-weighted imaging of breast tumors: quantitative and visual assessment. Japanese Journal of Radiology. 2011;29(6):429-36.

25. Bogner W, Gruber S, Pinker K, Grabner G, Stadlbauer A, Weber M, et al. Diffusion-weighted MR for Differentiation of Breast Lesions at 3.0 T: How Does Selection of Diffusion Protocols Affect Diagnosis? Radiology. 2009;253(2):341-51.

26. Tozaki M, Fukuma E. 1H MR Spectroscopy and Diffusion-Weighted Imaging of the Breast: Are They Useful Tools for Characterizing Breast Lesions Before Biopsy? American Journal of Roentgenology. 2009;193(3):840-9.

27. Wikipedia, contributors. Error function: Wikipedia, The Free Encyclopedia.; August 28, 2022. [updated November 22, 2023. Available from: <https://en.wikipedia.org/wiki/Error_function>.

28. Park MY, Byun JY. Understanding the mathematics involved in calculating apparent diffusion coefficient maps. AJR Am J Roentgenol. 2012;199(6):W784.

29. Liang J, Zeng S, Li Z, Kong Y, Meng T, Zhou C, et al. Intravoxel Incoherent Motion Diffusion-Weighted Imaging for Quantitative Differentiation of Breast Tumors: A Meta-Analysis. Frontiers in Oncology. 2020;10.
